# Out‐Of‐Plane Ordered Laminate Borides and Their 2D Ti‐Based Derivative from Chemical Exfoliation

**DOI:** 10.1002/adma.202008361

**Published:** 2021-08-05

**Authors:** Martin Dahlqvist, Jie Zhou, Ingemar Persson, Bilal Ahmed, Jun Lu, Joseph Halim, Quanzheng Tao, Justinas Palisaitis, Jimmy Thörnberg, Pernilla Helmer, Lars Hultman, Per O. Å. Persson, Johanna Rosen

**Affiliations:** ^1^ Thin Film Physics Division Department of Physics, Chemistry and Biology (IFM) Linköping University Linköping SE‐581 83 Sweden; ^2^ Materials Design Department of Physics, Chemistry and Biology (IFM) Linköping University Linköping SE‐581 83 Sweden

**Keywords:** 2D material, borides, chemical order, density functional theory, o‐MAB phase

## Abstract

Exploratory theoretical predictions in uncharted structural and compositional space are integral to materials discoveries. Inspired by *M*_5_SiB_2_ (T2) phases, the finding of a family of laminated quaternary metal borides, *M*′_4_ *M*″SiB_2_, with out‐of‐plane chemical order is reported here. 11 chemically ordered phases as well as 40 solid solutions, introducing four elements previously not observed in these borides are predicted. The predictions are experimentally verified for Ti_4_MoSiB_2_, establishing Ti as part of the T2 boride compositional space. Chemical exfoliation of Ti_4_MoSiB_2_ and select removal of Si and MoB_2_ sub‐layers is validated by derivation of a 2D material, TiO*_x_* Cl*_y_*, of high yield and in the form of delaminated sheets. These sheets have an experimentally determined direct band gap of ≈4.1 eV, and display characteristics suitable for supercapacitor applications. The results take the concept of chemical exfoliation beyond currently available 2D materials, and expands the envelope of 3D and 2D candidates, and their applications.

## Graphical abstract

Out‐of‐plane chemical order in layered T2 phase Ti_4_MoSiB_2_ (*o*‐MAB) is theoretically predicted and experimentally verified. Chemical exfoliation of the compound is shown by derivation of a 2D material, TiO*_x_* Cl*_y_*. The 2D sheets have a direct bandgap of ≈4.1 eV and display characteristics suitable for supercapacitor applications.

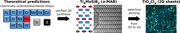

## 1 Introduction

The contemporary exploration of 2D materials is propelled by physical and chemical properties that are vastly different from their 3D parent phases. Since the discovery of graphene,^[^ ^1^ ^]^ the family of 2D materials has expanded and includes boron nitride (h‐BN),^[^ ^2^ ^]^ metal hydroxides,^[^ ^3^ ^]^ transitional metal dichalcogenides (TMDs),^[^ ^4^ ^]^ and oxides (TMOs),^[^ ^3^ ^]^ as well as more recently MXene carbides, nitrides, and carbonitrides.^[^ ^5^ ^]^ The uniqueness of 2D materials and range of surface functionalization offer opportunities for both fundamental and technological discoveries, and show potential for a wide range of applications, including energy storage, catalysis, superconductors, and carbon capture.^[^ ^6^, ^7^ ^]^

2D materials are exciting, where the composition and atomic arrangement play a defining role for the properties. A potential pathway for discovering new 2D materials is to start from a laminated 3D phase. The common approach is to exfoliate single or few atomic layers from such compounds with, for, strong in‐plane bonds and weak out‐of‐plane bonds. The exfoliation process is facilitated by mechanical force or ion‐exchange with osmotic swelling.^[^ ^1^, ^3^, ^8^ ^]^ This includes materials with van der Waals or hydrogen bonds between the layers, such as graphite, MoS_2_, h‐BN, and metal oxides. In particular, attention directed towards 2D metal oxides are spurred owing to their attractive functionalities, and being rich in both structural and chemical diversity as well as electronic properties.^[^ ^9^ ^]^ Their large number of possible oxidation states are advantageous for achieving large pseudocapacity^[^ ^8^ ^]^ combined with higher chemical stability than carbides and sulfides, which is desirable for enhanced durability in electrodes.^[^ ^10^ ^]^ Also, titanium oxide (TiO_2_) nanosheets have characteristics suitable for photocatalysis, and allows for layer‐by‐layer self‐assembly.^[^ ^11^ ^]^ Still, novel synthesis routes are desirable while keeping targeted functionality.

In addition to mechanical exfoliation, selective etching, also referred to as chemical exfoliation, has been proven as an alternative pathway for the synthesis of 2D materials from laminated parent 3D crystals with stronger interlayer interaction. A flagship example is the 2D MXenes,^[^ ^5^ ^]^ which is described by the generic formula of *M* *_n_*_+1_ *X* *_n_* *T* *_z_*, where *M* is an early transition metal, *X* is C and/or N, and *T* *_z_* denotes surface terminated functional groups, ‐O, ‐OH, ‐F, and Cl.^[^ ^12^, ^13^, ^14^ ^]^ MXenes are typically produced by the selective etching of the A‐group element, primarily Al, from the parent MAX phases, a large group of atomic laminates which comprises more than 150 members to date.^[^ ^15^ ^]^ Through selective etching of the *A* layers, experimental investigations have identified about 30 different MXenes, including alloy MXenes, showing high potential for applications ranging from energy storage and catalysis to electromagnetic interference shielding.^[^ ^6^, ^16^ ^]^ To date, all selectively etched 2D materials originate from MAX phases or related layered carbide materials.^[^ ^17^ ^]^ A few attempts have been reported for the synthesis of 2D borides through selective etching of layered MAB phases, like *M* *_n_*_+1_AlB_2_ *_n_* and *M*_4_AlB_4_, where M is a transition metal and *n* = 1–3, in HCl, HF or LiF‐HCl solutions.^[^ ^18^ ^]^ However, realization of individual single‐layer sheets has not been achieved to date, such that their characteristics remains to be explored.

Expanding the field of 2D materials requires identification of layered material candidates that can serve as a blueprint structure for conversion into 2D. One such potential layered material is the so‐called T2 phases with the general formula *M*_5_ *A*B_2_, where *M* is a transition metal (like Mo and Fe) and *A* is an A‐group element (Si, Ge, P). The T2 phase was discovered in 1957 when Nowotny et al. synthesized the prototypical Mo_5_SiB_2_^[^ ^19^ ^]^ followed by the synthesis of Fe_5_SiB_2_ and Mn_5_SiB_2_ phases in 1960.^[^ ^20^ ^]^ T2 is an atomically layered material and crystallizes in the tetragonal *I*4/*mcm* symmetry. Furthermore, the T2 phases are attractive due to their high oxidation resistance,^[^ ^21^ ^]^ nearly isotropic thermal expansion,^[^ ^22^ ^]^ and superconductivity.^[^ ^23^ ^]^ In addition, Fe_5_SiB_2_ and Fe_5_PB_2_ are uniaxial ferromagnets with earth‐abundant elements, and Co‐doping has been suggested to enhance the magnetocrystalline anisotropy energies.^[^ ^24^ ^]^

In this report we demonstrate an expansion of possible chemistries of the T2 phases by exploiting the two crystallographic unique metal sites upon metal alloying. Guided by theoretical predictions for thermodynamic and dynamic stability, we identify 11 thermodynamically stable quaternaries with out‐of‐plane chemical order, denoted *o*‐MAB phases. Out of these, we select Ti_4_MoSiB_2_ for experimental verification, and show chemical ordering in the form of alternating layers based on one metal element only. It should be noted that there is to date no reported T2 phase including Ti. Furthermore, molten ZnCl_2_ salt‐assisted chemical etching is utilized to transform the 3D Ti_4_MoSiB_2_ phase into 2D titanium oxychloride (TiO*_x_* Cl*_y_*) sheets. The as‐prepared 2D‐TiO*_x_* Cl*_y_* sheets exhibit semiconducting properties, with a direct bandgap of ≈4.1 eV, indicating potential applications in ultraviolet (UV) light detectors and for photocatalytic chemistry. Furthermore, the sheets are formulated into flexible electrodes displaying a substantial charge storage performance. Likewise important, the 2D TiO*_x_* Cl*_y_*‐based electrodes exhibit promising energy storage performance with a respectable volumetric capacitance of 275.2 F cm^–3^ at a scan rate of 5 mV s^–1^ and excellent rate capability.

## 2 Results

### 2.1 Theoretical Predictions

**Figure 1:**
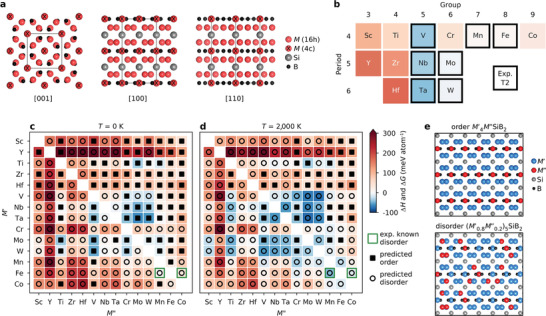
Thermodynamic stability maps of the ternary and quaternary T2 phases, colored to represent the order of lowest energy. a) Crystal structure of the T2 phase *M*_5_SiB_2_ along the [001], [100], and [110] zone axes, with *M*, Si, and B atoms represented by red, grey, and black dots, respectively. b) Calculated formation enthalpy ∆*H* for *M*_5_SiB_2_ with the experimentally reported T2 phases are marked by a black square. c,d) Calculated formation enthalpy ∆*H* and Gibbs free energy of formation ∆*G* at c) 0 K and d) 2000 K. The symbols represent the chemical order/solid solution of lowest energy for a given combination of *M*′ and *M*″, with a filled square for ordered *M*′_4_ *M*″SiB_2_ and an open circle for solid solution (*M*′_0.8_ *M*″_0.2_)_5_SiB_2_. e) Schematic representation of chemically ordered *M*′_4_ *M*″SiB_2_ (top) and solid solution (*M*′_0.8_ *M*″_0.2_)_5_SiB_2_ (bottom) projected along the [100] zone axis, with *M*′, *M*″, Si, and B atoms represented by blue, red, grey, black dots, respectively.

The T2 phases crystallize in the tetragonal *I*4/*mcm* symmetry, see **Figure** **1a**. The thermodynamic stability of the ternary *M*_5_SiB_2_ phases with *M* = Sc, Y, Ti, Zr, Hf, V, Nb, Ta, Cr, Mo, W, Mn, Fe, and Co is evaluated in Figure 1b, showing the calculated formation enthalpy Δ*H*_cp_ at 0 K, where blue region represents stable phases (Δ*H*_cp_ < 0). The corresponding identified equilibrium simplex for each phase is listed in Table S1, Supporting Information. Thermodynamically stable, or close to stable, M_5_SiB_2_ phases are found for *M* from Group 5 to 8 while *M*_5_SiB_2_ phases with *M* from Group 3 and 4 are far from stable, evident from the red areas. Experimentally known T2 phases are marked by a black square, and are all here identified as stable or close to stable (Δ*H*_cp_ < +10 meV atom^–1^), demonstrating that theory for predictive thermodynamic phase stability calculations of *M*_5_SiB_2_ phases is consistent with experiments.

Taking advantage of the two independent *M* sublattices in *M*_5_SiB_2_, Wyckoff sites 16l and 4c (Figure 1a), could potentially allow for chemical out‐of‐plane ordering when alloying between two metals, *M*′ and *M*″. A similar approach has recently been demonstrated for atomically layered carbides (*o*‐MAX phases).^[^ ^25^ ^]^ A schematic illustration of such ordered *M*′_4_ *M*″SiB_2_ structure and atomic coordination is shown in Figure S1, Supporting Information. To show whether chemical order or solid solution is preferred for the 182 unique combinations of *M*′ ≠ *M*′′ in *M*′_4_ *M*″SiB_2_, the thermodynamic stability is visualized in Figure 1c using a heatmap, where *M*′ and *M*″ are listed in the order of the periodic Group of the elements. The chemical order/solid solution of lowest energy is represented by filled squares for ordered *M*′_4_ *M*″SiB_2_ and open circles for solid solution *(M′_0.8_M*″_0.2_)_5_SiB_2_. The background color represents the calculated thermodynamic stability for the chemical configuration (ordered or solid solution) of lowest energy, with a blue region representing stable phases (Δ*H*_cp_ or Δ*G*_cp_ < 0). In addition, experimentally known quaternary solid solution (*M*′_0.8_ *M*″_0.2_)_5_SiB_2_ alloys are marked by a green square. The theoretically identified equilibrium simplex for all phases is listed in Table S2, Supporting Information. Figure 1c depicts the calculated formation enthalpy at 0 K, showing that 84 of the 182 *M*′ and *M*″ combinations are chemically ordered. Out of these, 22 are predicted thermodynamically stable, Δ*H*_cp_ < 0, with *M′ and M″* from Group 4 (Ti), 5 (V, Nb, Ta), and 6 (Cr, Mo, W). The reported quaternary solid solution phases, (Fe_0.8_Mn_0.2_)_5_SiB_2_ and (Fe_0.8_Co_0.2_)_5_SiB_2_,^[^ ^26^ ^]^ are predicted to have a chemically disordered configuration of lowest energy, though with Δ*H*_cp_ > 0.

Since materials synthesis is performed at *T* > 0 K, typically in the temperature range of 1000–1900 °C (1273–2173 K) for *M*_5_SiB_2_ and (Fe_0.8_ *M*″_0.2_)_5_SiB_2_ (*M*″ = Mn, Co), the contribution of configurational entropy to Gibb's free energy needs to be considered for solid solution (*M*′_0.8_ *M*″_0.2_)_5_SiB_2_. Herein, we choose to evaluate the contribution from configurational entropy at 2000 K. Figure 1d shows a heat map for 2000 K, obtained by comparing Δ*H*_cp_ of an ordered *M*′_4_ *M*″SiB_2_ with Δ*G*_cp_ of solid solution (*M*′_0.8_ *M*″_0.2_)_5_SiB_2_. The minimum of Δ*H*_cp_ or Δ*G*_cp_ represents the predicted stability (given by background color) and the predicted order (symbol) for a given combination of *M*′ and *M*″.

At elevated temperature, the number of *M′_4_M″*SiB_2_ predicted to be stable and ordered have been reduced from 22 to 11, while the number of stable solid solution (*M*′_0.8_ *M*″_0.2_)_5_SiB_2_ have increased to 42, including the experimentally reported (Fe_0.8_ *M*″_0.2_)_5_SiB_2_ (*M*″ = Mn, Co).^[^ ^26^ ^]^ In addition, several ordered and solid solution phases are found which are close to stable. The stable or close‐to‐stable phases are mainly discovered for *M*′ and *M*″ from Group 5 to 7, with a general preference for order where *M*″ = V, Cr, and Mn. Interestingly, we also predict that the chemically ordered Ti_4_MoSiB_2_ is stable, thermodynamically (Figure 1c,d) and dynamically (Figure S2, Supporting Information), despite the non‐existence of the hypothetical Ti_5_SiB_2_ (herein predicted to be not stable with Δ*H*_cp_ = +64 meV atom^–1^). This means that substitution of 80% of Mo atoms for Ti in the prototypical Mo_5_SiB_2_ phase is energetically preferred with respect to both solid solution (Ti_0.8_Mo_0.2_)_5_SiB_2_ and decomposition into other phases. Also note that the ternary Cr_5_SiB_2_ is predicted to be not stable, because of Δ*H*_cp_ = +44 meV atom^–1^. This is consistent with the lack of experimental reports of this phase, despite experiments in the concerned compositional domain, where instead the neighboring V_5_SiB_2_ and Mn_5_SiB_2_ have been realized (see Figure 1b). Figure 1d shows more examples where Cr can, in theory, be incorporated at the 4c‐site in the T2 phase with a preference for chemical order, such as for *M*′_4_CrSiB_2_ where *M*″ = Nb, Ta, Mo, and W. Preference for order is mainly governed by *M*′ being larger than *M*″ as shown in Figure S3, Supporting Information.

### 2.2 Out‐Of‐Plane Chemical Ordering in 3D Ti_4_MoSiB_2_ T2 Phase

**Figure 2:**
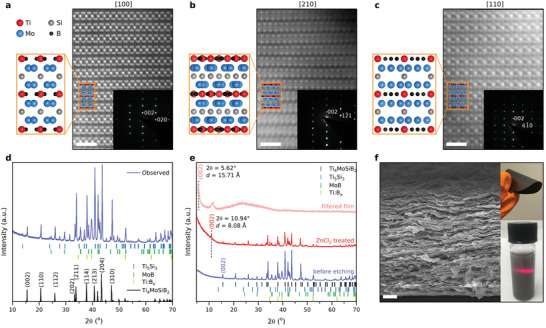
Characterization of synthesized Ti_4_MoSiB_2_. a–c) Out‐of‐plane chemical ordering of Ti_4_MoSiB_2_ evident from STEM images along the a) [100], b) [210], and c) [110] zone axes, respectively, with corresponding SAED patterns. Projected schematics to the left of each image represent the corresponding atomic arrangements predicted by DFT. The scale bar in (a–c) is 1 nm. d) XRD of sample with nominal composition Ti_4_MoSiB_2_ along with simulated diffractogram for ordered Ti_4_MoSiB_2_ and peak positions for secondary phases Ti_5_Si_3_, MoB, and Ti:B*_x_*, respectively. e) Simulated XRD peak positions for 3D Ti_4_MoSiB_2_ (black bars), XRD pattern of the Ti_4_MoSiB_2_ sample before (blue) and after (red) molten ZnCl_2_ etching, and after delamination and filtering (pink). f) SEM image showing a cross‐section of a 3.25 µm‐thick *d*‐TiO*_x_* Cl*_y_* “paper” (obtained after filtering), where the right inset shows a photograph of the flexible *d*‐TiO*_x_* Cl*_y_* “paper”, and the colloidal solution with dispersed 2D flakes (before filtering). The scale bar in (f) is 1 µm.

Guided by the theoretical predictions (Figure 1d) and challenging the present lack of Ti‐based T2 phases (Figure 1b), bulk synthesis was attempted for Ti_4_MoSiB_2_. The detailed synthesis process description is found in the methods section. Scanning transmission electron microscopy (STEM) images of the Ti_4_MoSiB_2_ T2 phase along the [100], [210], and [110] zone axes are shown in **Figure** **2a**‐c, respectively. For the applied imaging conditions, the Mo atoms appear brightest, the Ti and Si atoms are less bright, while B is too weak to be visible. An atomically laminated and chemically ordered atomic arrangement is evident along the [100] and [110] zone axes. The schematics at the left of each micrograph show the atomic arrangement predicted by DFT calculations, which is identical to the experimentally observed atomic arrangement. Additional STEM along [111] zone axis as shown in Figure S4, Supporting Information corroborates the ordered structure. The homogeneous elemental distribution of the Ti, Mo, and Si content within the material was verified by energy‐dispersive X‐ray (EDX) analysis, and is shown in Figure S5, Supporting Information, where the relative content of Ti, Mo, and Si is 63, 18, and 19 at%, respectively, which is close to the predicted ideal 4:1:1 molar ratio. In addition, the out‐of‐plane ordered structure is corroborated by the selective area electron diffraction (SAED) shown in the insets of Figure 2a‐c. For comparison, the simulated SAED for ordered Ti_4_MoSiB_2_, solid solution (Ti_0.8_Mo_0.2_)_5_SiB_2_, Ti_5_SiB_2_ and Mo_5_SiB_2_ are shown in Figure S6, Supporting Information, verifying the ordered Ti_4_MoSiB_2_ as the observed structure.

Figure 2d depicts the measured XRD pattern of the as‐prepared Ti_4_MoSiB_2_ sample, and the calculated diffractogram for Ti_4_MoSiB_2_ (≈40.6 wt%). Major impurity phases, that is, Ti_5_Si_3_ (17.5 wt%), MoB (24.2 wt%), and Ti:B*_x_* (17.7 wt%), are indicated with vertical lines. The Rietveld refinement and corresponding refinement parameters are shown in Figure S7 and Table S3, Supporting Information, respectively. The lattice parameters *a* and *c* calculated from Rietveld refinement are found to be 6.07 and 11.37 Å, respectively, consistent with (within 0.4%) the theoretically predicted parameters (Table S4, Supporting Information). Comparing the measured XRD pattern with simulated diffractograms from relaxed (using DFT) ordered Ti_4_MoSiB_2_, solid solution (Ti_0.8_Mo_0.2_)_5_SiB_2_, Ti_5_SiB_2_, and Mo_5_SiB_2_ further support the discovery of chemically out‐of‐plane ordered Ti_4_MoSiB_2_ (see Figure S8, Supporting Information).

The out‐plane chemical order identified in Ti_4_MoSiB_2_ takes advantage of the two distinct *M*‐sites, 16*l* and 4*c*, in the T2 phases. This concept has previously been utilized for creating out‐of‐plane order in MAX phases, the parent 3D material to the 2D MXene, hence being referred to as *o*‐MAX.^[^ ^27^ ^]^ In the same manner, we here choose to coin the out‐of‐plane ordered Ti_4_MoSiB_2_ as the first member of *o*‐MAB phases.

### 2.3 Derivation of 2D TiO*_x_* Cl*_y_* by Chemical Exfoliation

After determining the structure and composition of the chemically ordered Ti_4_MoSiB_2_, we explore the possibility of chemical exfoliation for derivation of the 2D counterpart. Figure 2e depicts the XRD patterns of as‐prepared Ti_4_MoSiB_2_ powder before and after etching in molten ZnCl_2_ salt, and of a filtered free‐standing film produced by intercalation with TBAOH with subsequent delamination in water. The peak intensities originating from the parent Ti_4_MoSiB_2_ crystal show an apparent decrease after immersion in molten ZnCl_2_ salt. As importantly, the (002) peak shifts from a 2θ equal to 15.65° down to 10.94°, which can be ascribed to an enlarged *d* spacing of 8.08 Å, from the original 5.69 Å (*c* lattice parameter/2). The additional low angle (002) peak is typical for most reported HF‐etched MXenes, and is also similar to that observed in recently reported Ti_3_C_2_Cl_2_ MXene from etching Ti_3_ZnC_2_ in molten ZnCl_2_.^[^ ^12^, ^13^ ^]^ Unlike the broad peaks typical of MXenes, the (00*l*) peaks of the herein etched derivative are relatively sharp and intense, indicating that the sheets are well aligned after etching. Moreover, the Ti_5_Si_3_, MoB, and Ti:B*_x_* secondary phases present in the scan of the Ti_4_MoSiB_2_ powder are found to be almost dissolved in the molten ZnCl_2_. After TBAOH treatment, the etched product is easily delaminated by mild sonication in water. The delaminated 2D sheets will henceforth be referred to as *d*‐TiO*_x_* Cl*_y_*.

The corresponding colloidal suspension (see inset in Figure 2f) was filtered through a nanoporous polypropylene membrane to obtain a free‐standing *d*‐TiO*_x_* Cl*_y_* film (see inset in Figure 2f). The filtered film has an intrinsic high flexibility without any additives. XRD (Figure 2e, topmost scan) shows two broad (00*l*) peaks, corresponding to a *d* spacing of 15.71 Å. The cross‐sectional morphology of the free‐standing film is shown in Figure 2f, wherein the stacked delaminated sheets are visible. The corresponding EDX (Figure S9, Supporting Information) analysis shows that Si is completely removed after the etching procedure, the Mo and B signals have decreased substantially to noise level, and the remaining film is composed of mainly Ti, O, and Cl.

**Figure 3:**
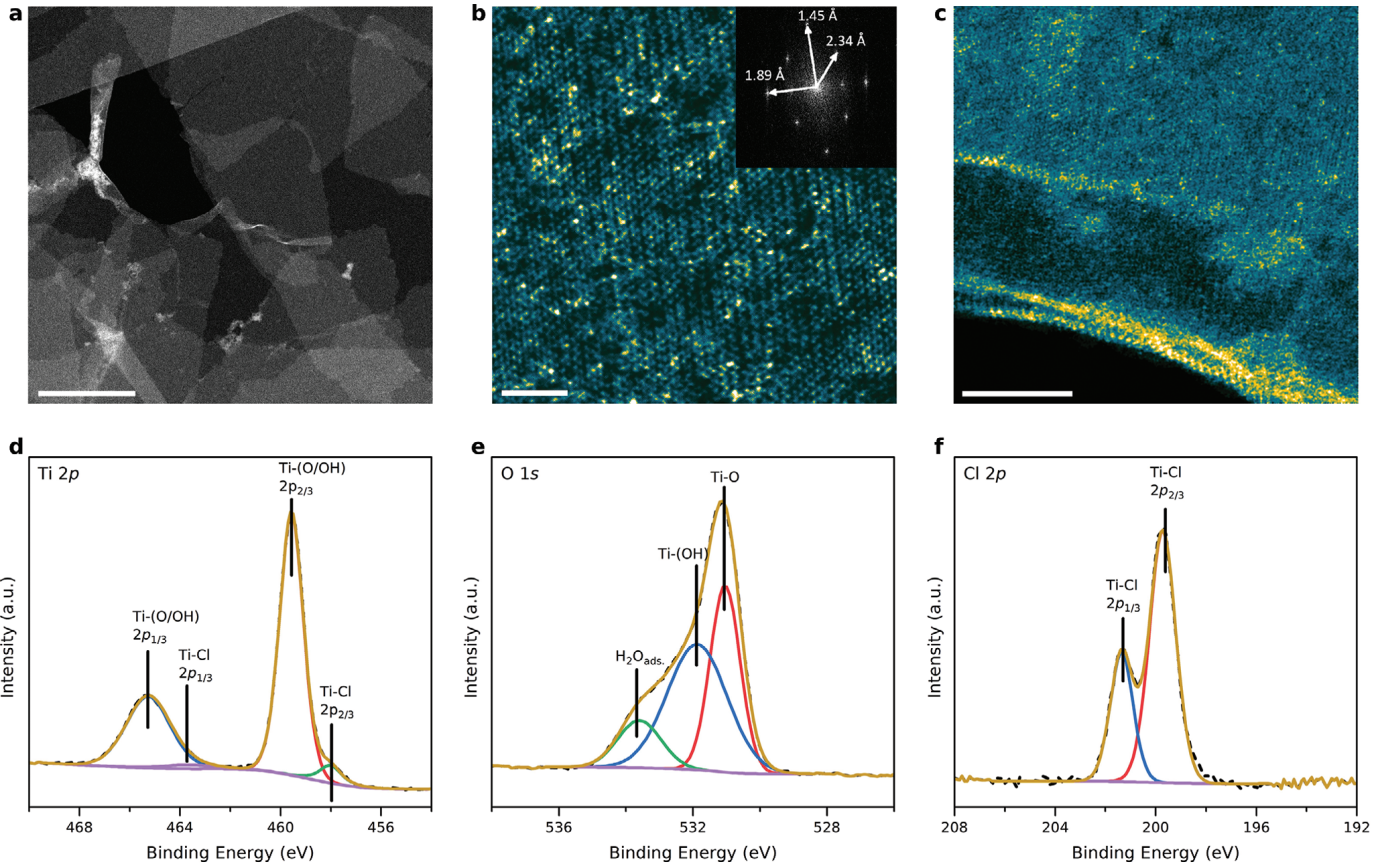
Morphological and structural characterizations of 2D TiO*_x_* Cl*_y_*. a) An overview STEM image showing several single‐layer flakes freestanding in vacuum. The scale bar in (a) is 100 nm. b) Atomically‐resolved STEM plan‐view image evidencing a tetragonal crystallinity, also highlighted in the FFT inset. The scale bar in (b) is 2 nm. c) Atomically‐resolved cross‐sectional STEM image if a folded sheet edge. The scale bar in (c) is 5 nm. d–f) High‐resolution XPS (defined in text) spectra with peak fittings for Ti 2*p*, O 1*s*, and Cl 2*p*, respectively.

To confirm the delamination into single sheets and their crystallinity, STEM was performed. **Figure** **3a** shows an overview STEM image of the delaminated sheets, where several single‐layer flakes can be observed, with an average sheet size of 0.1 µm^2^. At higher magnifications, as depicted in Figure 3b, the atomically resolved crystal structure of the flakes is presented together with the corresponding Fast Fourier Transform (FFT). The FFT reveals that the in‐plane lattice spacings, 1.89 ± 0.02 and 1.45±0.02 Å, respectively, are separated by an angle of 89.4°, indicating a tetragonal symmetry in this projection either slightly bent or with a minor off‐axis tilt with respect to the transmitted beam. Exfoliation into 2D atomic layered sheets is consequently confirmed, and is further demonstrated in Figure 3c, where sheets are found to bend locally and expose their cross‐sectional structure. It is further shown by electron energy‐loss spectroscopy (EELS) analysis that single flakes consists mainly of Ti, O, and Cl, together with very weak signals of Mo and B, and the molar ratio of Ti:O:Cl approximates 1:2.9:0.1 (Figure S10 and Table S5, Supporting Information).

XPS high resolution spectra for the Ti 2*p* region (Figure 2d, Table S7, Supporting Information) for i) Ti_4_MoSiB_2_ and ii) *d*‐TiO*_x_* Cl*_y_* (Figure S11, Supporting Information) show a shift in the binding energy (BE) of the Ti species; from 453.8 eV in Ti_4_MoSiB_2_ to 457.9 eV (Ti‐Cl) and 459.6 eV (Ti‐O/OH) in *d*‐TiO*_x_* Cl*_y_*. This shift is due to the substitution of Mo, Si, and B for O and Cl during etching. The BE of the Ti‐O species in *d*‐TiO*_x_* Cl*_y_* is 0.6 eV higher than that for TiO_2_, indicating that there is a difference in the Ti‐O/OH bonding between TiO_2_ and *d*‐TiO*_x_* Cl*_y_* which arises from the change in dimensionality from 3D to 2D and presence of both O and OH surface terminations. Similarly, for the O 1s region (Figure 3e, Figure S12d and Table S11, Supporting Information), the BE of the O species belonging to the metal oxides in Ti_4_MoSiB_2_ (530.5 eV) is 0.6 eV lower than that for Ti‐O in *d*‐TiO*_x_* Cl*_y_*. This shift indicates that there is a difference in Ti‐O bond in *d*‐TiO*_x_* Cl*_y_* as compared to 3D TiO_2_ which, as stated above, can be related to the change in dimensionality from 3D to 2D. Figure 3f presents the high‐resolution spectra of the Cl 2p region for *d*‐TiO*_x_* Cl*_y_*, showing Ti‐Cl species at BE of 199.7, 1.7 eV higher than that for TiCl_4_. Altogether, the chemical formula of *d*‐TiO*_x_* Cl*_y_* from XPS is calculated to be TiO_1.4±0.1_(OH)_0.9±0.24_Cl_0.2±0.05_⋅(0.35±0.15)H_2_O_ads._, where H_2_O_ads._ is adsorbed water on the surface of the free‐standing film or intercalated water between the layers, considering the moles of Ti as the base equal to 1. We attribute the O, OH, and Cl in the formula as surface terminating species. From the chemical formula we can estimate the oxidation state for Ti to be +(3.9 ± 0.6). Despite differences in global versus local probing, the composition of the sheets matches the EELS result with a minor discrepancy (within error bars). Details of the XPS analysis are found in the Supplementary materials (Figures S11–S12, Tables S6–S12, Supporting Information).

Based on the aforementioned results, it is reasonable to suggest that the Si layer in Ti_4_MoSiB_2_ was selectively etched when immersed in molten ZnCl_2_, with a possible reaction between the Mo‐B layer and ZnCl_2_ occurring simultaneously. The latter is supported by MoCl_3_ decomposing at about 410 °C (the etching herein is performed at 600 °C) and BCl_3_ being a volatile gas phase with a boiling point of 12.6 °C, which explains the absence of these elements in any observed etching products. The exposed Ti atoms were bonded with oxygen and chlorine. In the subsequent dilute HCl and water washing process, a certain amount of Cl‐terminations may be further replaced by O‐containing moieties. The chemical reactions occurring during the etching process of Ti_4_MoSiB_2_ can therefore be described by the following simplified equations:

(1) $$\begin{array}{c} {\text{Ti}}_{4}{\text{MoSiB}}_{2}+2{\text{ZnCl}}_{2}={\text{Ti}}_{4}-[\text{MoB}]+{\text{SiCl}}_{4}\left(g\right)+2\text{Zn} \end{array}$$

(2)

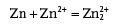

(3) $$\begin{array}{c} {\text{Ti}}_{4}-\text{[MoB]}\;+\;{\text{8Cl}}^{-}={\text{Ti}}_{4}{\text{Cl}}_{2}\;+\;{\text{MoCl}}_{3}\;+\;{\text{BCl}}_{3}\;+{\text{ 8e}}^{-} \end{array}$$

(4) $$\begin{array}{c} 4{\text{Zn}}^{2+}+8e^{-}=4\text{Zn} \end{array}$$

(5) $$Ti_{4}{\text{Cl}}_{2}+H_{2}O={\text{TiO}}_{x}{\text{Cl}}_{y}+H_{2}$$

Since the silicon layer is presumably weakly bonded to the Ti atoms in the Ti_4_MoSiB_2_ sublayer, it can be easily oxidized into a Si^4+^ cation by Lewis acidic Zn^2+^ and then spontaneously bond with the intercalated Cl^–^ to form a volatile SiCl_4_ with a boiling point of 57.65 °C, accompanied by the reduction of Zn^2+^ into zinc metal.^[^ ^12^ ^]^ Since excessive amount of ZnCl_2_ was used during the etching, the newly formed zinc metal would re‐dissolved in molten ZnCl_2_ through a redox reaction between Zn and Zn^2+^, which explains that only a small amount of zinc metal was detected in the final product. A similar behavior has been observed in the formation process of Ti_3_C_2_Cl_2_ MXene through etching of Ti_3_AlC_2_ MAX phase in molten ZnCl_2_ salt,^[^ ^13^ ^]^ and the role of Zn^2+^ and Cl^–^ in molten ZnCl_2_ in our case is analog to that of H^+^ and F^–^ in an aqueous HF solution.

### 2.4 Optical and Charge Storage Properties of 2D TiO*_x_* Cl*_y_*

**Figure 4:**
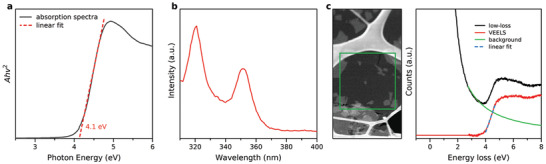
Optical properties of 2D TiO*_x_* Cl*_y_* sheets. a) Absorption and b) fluorescence spectra of TiO*_x_* Cl*_y_* excited at 270 nm. c) Monochromated STEM‐EELS spectrum image of a 2D TiO*_x_* Cl*_y_* sheets (green square) and the corresponding valence EELS (VEELS) spectra (red) including the ZLP (black) and the background‐subtracted VEELS data (green).

While the color of the 2D TiO*_x_* Cl*_y_* colloidal solution is medium gray, which is lighter than MXene solutions of a typical black color, we further investigated the optical properties of the 2D TiO*_x_* Cl*_y_* sheets (see **Figure** **4a**–c). According to the collected Ultraviolet‐visible (UV–vis) absorption spectrum of the dilute solution (0.033 mg mL^−1^), the bandgap energy of TiO*_x_* Cl*_y_* sheets was estimated to be 4.1 eV. The corresponding photoluminescence (PL) spectrum is shown in Figure 4b, where the 2D TiO*_x_* Cl*_y_* sheets exhibit an excitation‐dependent photoluminescence behavior. The PL spectrum excited by 285 nm light is consistent with the absorption spectrum (Figure S13, Supporting Information). When excited by 316 nm wavelength light, a second PL peak was detected, located at 355 nm, which could stem from deep energy level transitions.^[^ ^28^ ^]^ To exclude possible contamination effects, a filtered free‐standing film of the 2D TiO*_x_* Cl*_y_* was tested as well, and its photoluminescence behavior, shown in Figure S14, Supporting Information, is consistent with those of the colloidal solution in Figure 4b.

For a spatially resolved measurement of the optical properties, monochromated STEM‐EELS was further performed on a single 2D TiO*_x_* Cl*_y_* sheet (see Figure 4c). A spectrum image was recorded in the indicated area, and the low loss spectrum was averaged from spectra acquired on the single sheet, to remove the signal from the carbon grid, residues and multiple sheets. The averaged spectrum was subsequently background subtracted using a power‐law function and the single sheet spectrum is shown together with a linear fit that reveals a bandgap of ≈4.1 eV, in agreement with the optical measurements.

The potential of the present 2D TiO*_x_* Cl*_y_* in energy storage applications is assessed by constructing a three‐electrode Swagelok cell, where 2D *d*‐TiO*_x_* Cl*_y_* “paper”, activated carbon and Ag/AgCl served as a working, counter, and reference electrode, respectively. The filtered 2D TiO*_x_* Cl*_y_*‐based free‐standing film shows high flexibility and, when used as a capacitive electrode, renders a volumetric capacitance of 275 F cm^–3^ at 5 mV s^–1^ and excellent rate capability (185.9 F cm^–3^ at 1000 mV s^–1^), see Figure S15, Supporting Information. Details for the electrochemistry characterization is found in Section S3, Supporting Information.

## 3 Conclusions

MXenes, obtained from selective removal of specific atomic layers in the parent MAX phases, were discovered in 2011. Since then more than 30 MXenes have been experimentally realized. The selectively etched 2D material presented herein, TiO*_x_* Cl*_y_*, from Ti_4_MoSiB_2_ provides evidence that the concept of selective etching can be expanded beyond MAX/MXene to laminated borides. Considering the vast amount and abundant chemistry of known as well as here predicted laminated borides, this is a discovery which has far reaching implications with respect to novel 3D materials and their 2D derivatives, and their applications. Also considering the large number of known laminated 3D materials, beyond MAX‐related materials and borides, we suggest that the structural and composition space of 2D materials from selective etching can be drastically increased. In addition to this: With alloying comes the possibility for property tuning, and to include elements not usually present in the structures and compositions known to date. This is of importance for the applicability of both the 3D and 2D materials.

In conclusion, an expansion of attainable compositions of the T2 phases, that is, *M*_5_ *A*B_2_, was theoretically demonstrated by exploiting the two crystallographic unique metal (*M*) sites upon metal alloying, under the constraints of both chemical order and solid solution. The list of predicted stable quaternary T2 phases amounted to 11 ordered and 42 solid solutions, altogether expanding the elemental space of these borides with four elements (Sc, Ti, Hf, Cr) not previously known in the ternary counterparts. These predictions were experimentally confirmed by introducing Ti into the elemental space of the T2 phases, through synthesis the out‐of‐plane chemically ordered Ti_4_MoSiB_2_, which we propose is the first member in a family that we coin *o*‐MAB phases. Selective etching of Si and MoB sublayers from the parent Ti_4_MoSiB_2_ compound in ZnCl_2_ molten salt resulted in single layer sheets of the 2D derivative TiO*_x_* Cl*_y_*. This 2D material is a semiconductor with an experimentally measured direct band gap of ≈4.1 eV. Furthermore, a filtered 2D TiO*_x_* Cl*_y_*‐based free‐standing film shows high flexibility and, when used as a capacitive electrode, indicate the potential of using 2D *d*‐TiO*_x_* Cl*_y_* nanosheets in flexible supercapacitors. Altogether, based on the results presented herein, we suggest novel laminated borides materials which can be derived into their 2D counterparts. This is of importance for attainable properties and their use in applications.

## 4 Experimental Section

### Theoretical Details

All first‐principles calculations were performed by means of density functional theory (DFT) and the projector augmented wave method,^[^ ^29^ ^]^ as implemented within the Vienna ab‐initio simulation package (VASP) version 5.4.1.^[^ ^30^ ^]^ The generalized gradient approximation (GGA) as parameterized by Perdew–Burke–Ernzerhof (PBE)^[^ ^31^ ^]^ was used for treating the electron exchange and correlation effects. For phases with Cr, Mn, Fe, and Co, the spin‐polarized GGA version with multiple spin configurations considered was used. While the present study involves use of GGA for predictions of phase stability, it should be noted that predictions of electronic properties for *d*‐metal compounds based on, for example, Fe and Co and in particular metal oxides, could be improved by using of other functionals such as Hubbard U.^[^ ^32^ ^]^ Presented results are for the lowest energy spin configuration. A plane‐wave energy cut‐off of 400 eV was used and the Brillouin zone was integrated by Monkhorst–Pack special k‐point sampling,^[^ ^33^ ^]^ with a density of 0.05 Å^–1^. The total energy is minimized through relaxation of unit‐cell shape and volume, and internal atomic positions until satisfying an energy convergence of 10^–5^ eV atom^−1^ and force convergence of 10^–2^ eV Å^–1^.

In this work *M*′ and *M*″ from Group 3 to 9 were considered; Sc, Y, Ti, Zr, Hf, V, Nb, Ta, Cr, Mo, W, Mn, Fe, and Co. Chemical order of *M*′ and *M*″ have been modelled by taking advantage of the two different *M* sublattices in the prototypical Mo_5_SiB_2_ structure; Wyckoff sites 16*l* occupied by *M*′ and 4*c* by *M*″. In addition, chemical disorder of *M*′ and *M*″, that is, solid solution, have been modelled on the two *M* sublattices simultaneously using the special quasi‐random structure (SQS) method^[^ ^34^ ^]^ with supercell sizes up to 320 atoms. Convergency tests show that the supercells used give qualitative accurate representation and quantitative convergency in terms of calculated formation enthalpies, equilibrium volumes, and lattice parameters. The compositional notation used to indicate order or solid solution throughout this work is *M*′_4_ *M*″SiB_2_ for order and (*M*′_0.8_ *M*″_0.2_)_5_SiB_2_ for solid solution.

The thermodynamic stability has been investigated at 0 K with respect to decomposition into any combination of competing phases. The most competing set of competing phases, denoted equilibrium simplex, is identified using a linear optimization procedure^[^ ^35^, ^36^ ^]^ which have been proven successful to confirm already experimentally materials as well as predicting the existence of new ones.^[^ ^36^, ^37^ ^]^ The stability of a phase is quantified in terms of the formation enthalpy Δ*H*_cp_ by comparing its energy to the energy of the equilibrium simplex according to

(6) $$\begin{array}{c} \Delta H_{\text{cp}}=E\left(\text{MAB}\right)-E\left(\text{equilibrium}\;\;\text{simplex}\right) \end{array}$$

A phase is concluded stable when Δ*H*_cp_ < 0. Here *E*(MAB) represent the chemical order of lowest energy being chemically ordered *M*′_4_ *M*″SiB_2_ or solid solution (*M*′_0.8_ *M*″_0.2_)_5_SiB_2_. However, when *T* ≠ 0 K, the contribution from configurational entropy resulting from a solid solution distribution of *M*′ and *M*″ on the *M* sublattices in (*M*′_0.8_ *M*″_0.2_)_5_SiB_2_ will decrease the Gibbs free energy $\Delta G_{\text{cp}}^{\text{solid}\;\;\text{solution}}$ as approximated by

(7) $$\begin{array}{c} \Delta G_{cp}^{\text{solid}\;\;\text{solution}}[T]=\Delta H_{cp}^{\text{solid}\;\;\text{solution}}-T\Delta S \end{array}$$

where the entropic contribution Δ*S*, assuming an ideal solution of *M*′ and *M*″ on the *M*‐sites, is given by

(8) $$\begin{array}{c} \Delta S=-2k_{B}\left[x\ln\left(x\right)+\left(1-x\right)\ln\left(1-x\right)\right] \end{array}$$

where *k*_B_ is the Boltzmann constant and *x* is the concentration of *M*″ on the *M*‐sublattices.

Dynamical stability was evaluated by phonon calculations using the finite displacement method, as implemented in the PHONOPY code,^[^ ^38^ ^]^ with displacements of 0.01 Å used for calculations of the force constants. Calculations were performed using a 3 × 3 × 2 supercell with a *k*‐point sampling of at least ten points per Å^–1^, resulting in a 4 × 4 × 3 *k*‐point mesh. The calculations were converged to at least 10^–9^ eV atom^−1^ and 10^–3^ eV Å^−2^.

### Synthesis of Ti_4_MoSiB_2_ T2 Phase

The Ti_4_MoSiB_2_ powders were prepared by solid‐state reaction sintering of Ti/Mo/Si/B powder mixtures in tube furnace. In detail, the as‐received commercially elemental powders (boron (99.999%) and Mo (99.99%) from Sigma‐Aldrich, Si (99.8%), and Ti (99.99%) from Alfa Aesar) were weighed with a stoichiometric molar ratio and mixed thoroughly in an agate mortar. The resulting homogenous mixture was poured into an alumina crucible and put into the tube furnace, and then heated to 1500 °C for 120 min in an Ar atmosphere. After cooling to room temperature, loosely sintered sample was crushed using mortar and pestle, then grind and sieved through a 450‐mesh screen.

### Synthesis of 2D TiO*_x_* Cl*_y_*

Ti_4_MoSiB_2_ boride precursor and ZnCl_2_ (98%, Sigma‐Aldrich) with appropriate molar ratio (1:10) were mixed thoroughly and cold pressed into a disk with a diameter of 1 cm. The disk was then sealed in a quartz tube under vacuum, and the reaction were taken place at 600 °C in molten ZnCl_2_ (melting point: 290 °C) for 8 h. After cooled down to room temperature, the mixture was immersed with dilute HCl (37%, 1.18 kg L^–1^, VWR Chemicals) solution (0.5 m) to remove Zn metal, and then washed with degassed deionized water for several times to remove residual salts, and finally filtered with microfiltration membrane to separate the powders. The obtained powders were dried under vacuum at room temperature for 48 h. The dried powder was kept for further experiments and characterization. The estimated overall yield is 70%. For delamination, 0.3 g of the etched powder and 10 mL tetrabutyl ammonium hydroxide (TBAOH) (40 wt% in H_2_O, Sigma‐Aldrich) were added to a centrifuge tube, which was shaken manually for about 10 min. The tube was then centrifuged at 5500 rpm. for 4 min to remove the supernatant. Water was added to the tube to wash away the remaining TBAOH, after which the water was poured out. The process was repeated five times. Attention should be paid to avoid agitating and shaking at this step, in order to prevent the delamination. Finally, 30 mL water was added to the powder and shaken for about 3 min, and then sonicated under N_2_ bubbling about 1 h for delamination into single‐ or few layered 2D sheets with a concentration of 0.4–0.5 mg mL^–1^.

### Preparation of Free‐Standing *d*‐TiO*_x_* Cl*_y_* film

Free‐standing *d*‐TiO*_x_* Cl*_y_* film was prepared from 30 mL water mixture containing *d*‐TiO*_x_* Cl*_y_* flakes obtained above. The mixture was then centrifuged for 10 min at 3000 rpm, and the supernatant was collected. The colloidal suspension was finally filtered with nanoporous polypropylene membranes. The free‐standing *d*‐TiO*_x_* Cl*_y_* film can be peeled off easily from the membrane, and film with varied thickness can be prepared. For optical properties measurement, the free‐standing film was dried in a vacuum oven at room temperature for 48 h. For electrochemical performance test, the free‐standing film was used immediately after the filtration without over‐drying.

### Materials Characterization

XRD measurements of the Ti_4_MoSiB_2_ powders, etched product, intercalated sample, and *d*‐TiO*_x_* Cl*_y_* film were carried out on a diffractometer (Panalytical X'pert). The XRD scan of the Ti_4_MoSiB_2_ powders was analyzed by the Rietveld refinement method, using the FULLPROF code. The microstructure and chemical composition were observed by Scanning Electron Microscope, SEM (LEO 1550 Gemini) coupled with an energy dispersive spectrometer (EDS). For the out‐of‐plane ordered Ti_4_MoSiB_2_ T2 phase and corresponding 2D TiO*_x_* Cl*_y_* sheets, high‐angle annular dark‐field (HAADF) STEM imaging, EDX and EELS analysis were performed using a double‐corrected FEI Titan^3^ S/TEM, operated at 300 and 60 kV, respectively equipped with a FEI STEM detector and a Super‐X EDX detector. Selective area electron diffraction (SAED) was performed on a FEI Tecnai G2 TEM operated at 200 kV. 2D TiO*_x_* Cl*_y_* samples were prepared by drop‐casting 0.1 µL single‐flake‐solution on a DENSsolutions through‐hole Wildfire nanochip and placed in a DENSsolutions Wildfire double‐tilt heating holder. Atomic resolution STEM‐HAADF images were acquired after in situ heating in vacuum to 350 °C for 1 h to remove surface contamination. Low‐dose (≈10 pA probe current) imaging was performed (to preserve the 2D structure) at an optimized 21.5 mrad convergence angle, which provides a ≈0.7 Å probe at 300 kV. EELS spectra were collected in STEM‐dual EELS mode over a several microns large area at an accelerating voltage of 300 kV by employing a Gatan GIF Quantum ERS imaging filter and using a probe current slightly above 100 pA. Quantification was performed via built‐in functions in Gatan Digital Micrograph. VEELS spectra were collected in monochromated STEM mode at 60 kV acceleration and with a 1.4 V excitation potential of a FEI Wien‐type monochromator. The energy resolution was measured to 80 meV over 0.05 s acquisitions at 0.01 eV dispersion using a convergence semi‐angle of 21.5 mrad and a collection angle of 7.2 mrad. ZLP background subtraction was carried out using a pre‐measured ZLP in vacuum under identical conditions.

XPS measurements were performed on cold pressed disc of MAB (Ti_4_MoSiB_2_) and free‐standing film of *d*‐TiO*_x_* Cl*_y_* sample using a surface analysis system (Kratos AXIS Ultra^DLD^, Manchester, U.K.) using monochromatic Al‐Kα (1486.6 eV) radiation. The X‐ray beam irradiated the surface of the sample at an angle of 45°, with respect to the surface and provided an X‐ray spot of 300 × 800 µm. Charge neutralization was performed using a co‐axial, low energy (≈0.1 eV) electron flood source to avoid shifts in the recorded binding energy, BE. XPS spectra were recorded for Ti 2p, Mo 3d, Si 2p, B 1s, and O 1s, (not shown here but used for elemental quantification are the following regions: C 1s, Cl 2p, Zn 2p, and N 1s). Nitrogen in the free‐standing *d*‐TiO*_x_* Cl*_y_* film originates from the intercalation with TBAOH which is used for delamination. The analyzer pass energy used for all the regions was 20 eV with a step size of 0.1 eV. The BE scale of all XPS spectra was referenced to the Fermi‐edge (*E*_F_), which was set to a BE of zero eV in accordance with ref. ^[^ ^39^ ^]^. The peak fitting was carried out using CasaXPS Version 2.3.16 RP 1.6 and the global elemental percentage was quantified in the same manner as in refs.^[^ ^14^, ^40^ ^]^ as well as the calculation of the chemical formula for the *d*‐TiO*_x_* Cl*_y_* free standing thin film. The peak fitting for the Ti 2p region of the *d*‐TiO*_x_* Cl*_y_* free standing thin film was done using a Shirely background while keeping the FWHM for the peaks belonging to Ti‐(O/OH) and Ti‐Cl species within a margin of ±0.1 eV, the distance between the Ti 2p_2/3_ and Ti 2p_1/3_ for both species is also kept within a margin of ±0.1 eV and the ratio between Ti 2p_2/3_ and Ti 2p_1/3_ was fixed to 2:1. For the same sample, the peak fitting for the O 1s region was performed such that the area of the peak belonging to Ti‐O was forced to be kept at the minimum value while maintaining the minimum standard residual error, in order for the oxidation state of Ti to not exceed +4. A Shimadzu UV‐2450 Spectrophotometer is used for absorption spectroscopy. A Horiba Jobin Yvon Fluoromax‐4 spectra fluorometer is used for photoluminescence spectroscopy.

### Electrochemical Characterization

The electrochemical performance has been evaluated using a three‐electrode setup, where free‐standing *d*‐TiO*_x_* Cl*_y_* film, saturated Ag/AgCl and activated carbon served as active electrode, reference electrode and counter electrode, respectively. 1 m H_2_SO_4_ was used as an electrolyte. The active material loading was ≈ 1.41 mg cm^–2^ and the thickness of free‐standing *d*‐TiO*_x_* Cl*_y_* film was 3.25 µm. The cyclic voltammetry (CV) was carried out in the voltage range of ‐0.1 to +0.4 V (vs Ag/AgCl) at different scan rates, ranging from 5 to 1000 mV s^–1^. The galvanostatic charge/discharge measurements were conducted in the same voltage range at different current densities, ranging from 1 to 10 A g^–1^. The volumetric capacitance was calculated by using the given equation:

(9) $$\begin{array}{c} C_{\text{vol}}=\frac{1}{2}\frac{A}{Vsv} \end{array}$$

where *A* corresponds to the area of the CV curve at a given scan rate, *V* refers to the voltage window (0.5 V), *s* represents the scan rate, and *v* denotes the volume of working electrode. A glassy carbon electrode, which has a diameter of 3 mm was used, that is, an electrode size that is standard for glassy carbon electrode measurements was used. Altogether, a mass of 100 µg was used. The data has been reproduced four times, with material from different batches.

## Conflict of Interest

The authors declare no conflict of interest.

## Supporting information

Supporting Information

## Data Availability

The data that support the findings of this study are available from the corresponding author upon reasonable request.
