# Supplementary material for: Out‐Of‐Plane Ordered Laminate Borides and Their 2D Ti‐Based Derivative from Chemical Exfoliation
Source: Adv Mater. 2021 Aug 5;33(38):2008361. doi: 10.1002/adma.202008361 (PMC11468983; doi:10.1002/adma.202008361)
Supplement: Supplementary file 1 — Supporting Information [file ADMA-33-2008361-s001.pdf]

© 2021 Wiley-VCH GmbH

ADVANCED MATERIALS

Supporting Information

for *Adv. Mater.,* DOI: 10.1002/adma.202008361

Out-Of-Plane Ordered Laminate Borides and Their 2D Ti-Based Derivative from Chemical Exfoliation

Martin Dahlqvist, Jie Zhou,* Ingemar Persson, Bilal Ahmed, Jun Lu, Joseph Halim, Quanzheng Tao, Justinas Palisaitis, Jimmy Thörnberg, Pernilla Helmer, Lars Hultman, Per O. Å. Persson, and Johanna Rosen**

Supporting Information

Out-of-plane ordered laminate borides and their two-dimensional Ti-based derivative from chemical exfoliation

Martin Dahlqvist, Jie Zhou,*, Ingemar Persson, Bilal Ahmed, Jun Lu, Joseph Halim, Quanzheng Tao, Justinas Palisaitis, Jimmy Thörnberg, Pernilla Helmer, Lars Hultman, Per O. Å. Persson, Johanna Rosen**

* corresponding authors: martin.dahlqvist@liu.se; jie.zhou@liu.se; johanna.rosen@liu.se

S1. Crystal structure and calculated stability of M5SiB2

Table S1. Calculated formation enthalpy ΔH_{cp} (in meV atom^{-1}) and identified equilibrium simplex for M_5SiB_2 phases. Experimentally known M_5SiB_2 phases are marked in bold.

M	ΔH_{cp} (meV atom^{-1})	Equilibrium simplex	Synthesis reference
Sc	105	Sc, ScB_2, Sc_5Si_3	
Y	163	Y, YB_2, Y_5Si_3	
Ti	64	TiB, Ti_6Si_2B, Ti	
Zr	135	Zr, Zr_2Si, ZrB_2	
Hf	171	Hf, HfB_2, Hf_2Si	
**V**	-38	V_3B_2, V_5Si_3, V_3Si	[1, 2]
**Nb**	-34	Nb_3B_2, Nb_5Si_3, Nb	[3]
**Ta**	-42	Ta_2Si, Ta_3B_2	[1, 4]
Cr	44	CrB, Cr_3Si	
**Mo**	-3	MoB, Mo_3Si	[5, 6]
**W**	-5	W_2B, WSi_2, W	[5, 7]
**Mn**	7	Mn_2B, MnSi	[8, 9]
**Fe**	6	Fe_2B, FeSi	[8, 10]
Co	29	CoB, Co_2Si, Co	

The T2-phase has a Cr_5B_3-type tetragonal cell, shown in Figure S1, with 32 atoms per unit cell (space group *I*4/*mcm*) distributed on four sublattices with Wyckoff positions: *M*´ = 16l, *M*´´ = 4c, Si = 4a, B = 8h. The structure of T2 can be divide into three different layers where layer 1 consists of *M*´´ and B; layer 2 only of *M*´, and layer 3 by Si atoms. An overview of the structure is shown in Figure S2. The *M*´´(4c) atoms have coordination number 14: four B atoms in the plane; four *M*´ atoms in the plane above and four *M*´ atoms in the plane below; one Si above and one below. The *M*´´(16l) atoms in layer 2 have coordination number 9: two *M* atoms in the plane; two *M* and two B atoms in the neighboring plane; two *M* atoms and one Si atom in the plane below.

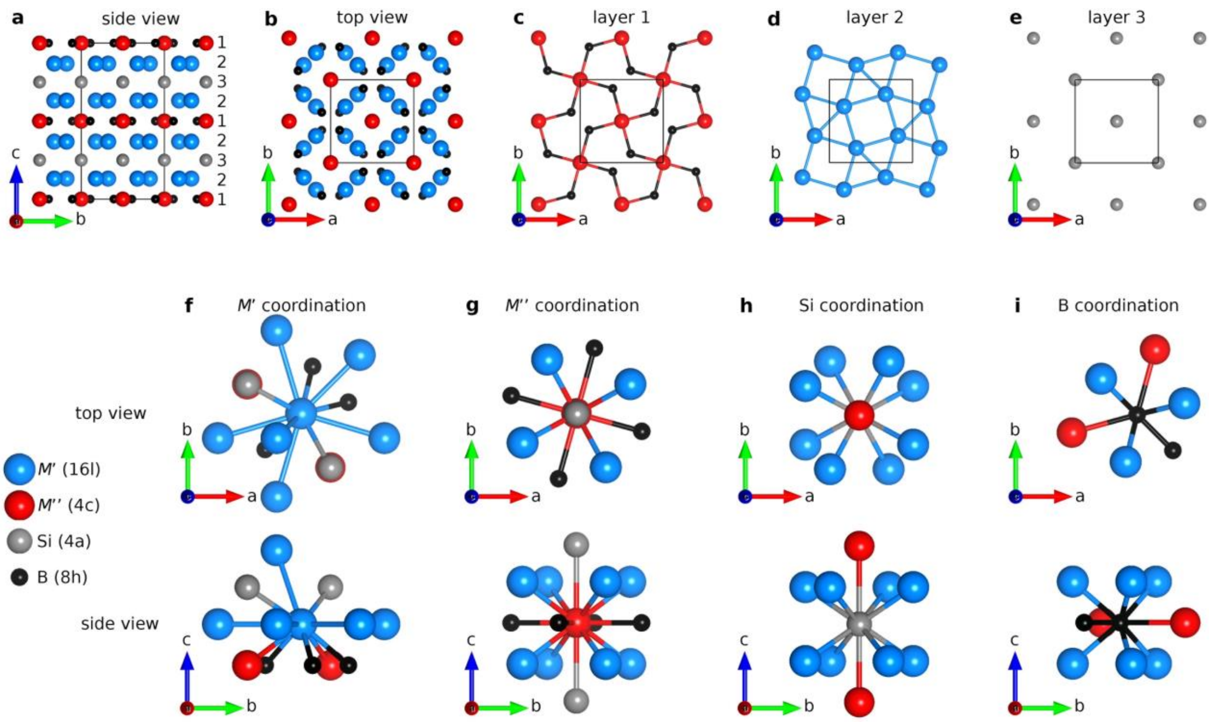

Figure S1. a,b) Schematic overview of the M'_4M''SiB$_2$ structure and. c-e) Structure of individual layers, as marked in (a), along the [001] zone axis. Unit cell is given by black box. The coordination for *M´* (f), *M´´* (g), Si (h) and B (i).

Table S2. Identified equilibrium simplex for *M´*:*M´´*:Si:B at the 4:1:1:2 composition.

M´	*M´´*	Equilibrium simplex
Sc	Y	Sc, Y, ScB_2, Sc_5Si_3
Sc	Ti	Sc, TiB_2, Sc_5Si_3
Sc	Zr	Sc, ZrB_2, Sc_5Si_3
Sc	Hf	Sc, HfB_2, Sc_5Si_3
Sc	V	Sc, VB, ScB_2, Sc_5Si_3
Sc	Nb	Sc, NbB, ScB_2, Sc_5Si_3
Sc	Ta	Sc, TaB, ScB_2, Sc_5Si_3
Sc	Cr	Sc, Cr, ScB_2, Sc_5Si_3
Sc	Mo	Sc, ScB_2, Mo, Sc5Si3
Sc	W	Sc, W, ScB_2, Sc_5Si_3
Sc	Mn	Sc, Mn, ScB_2, Sc_5Si_3
Sc	Fe	Sc, Fe, ScB_2, Sc_5Si_3
Sc	Co	Sc, ScB_2, Co, Sc_5Si_3
Y	Sc	Y, ScB_2, Y_5Si_3
Y	Ti	Y, TiB_2, Y_5Si_3
Y	Zr	Y, ZrB_2, Y_5Si_3
Y	Hf	Y, HfB_2, Y_5Si_3
Y	V	Y, VB, Y_5Si_3
Y	Nb	Y, NbB, Y_5Si_3
Y	Ta	Y, TaB, YB_2, Y_5Si_3
Y	Cr	Y, CrB, YB_2, Y_5Si_3
Y	Mo	Y, MoB, YB_2, Y_5Si_3
Y	W	Y, YB_2, W, Y_5Si_3
Y	Mn	Y, YB_2, Mn_2B, Y_5Si_3
Y	Fe	Y, YB_2, Fe_2B, Y_5Si_3
Y	Co	Y, Co, YB_2, Y_5Si_3
Ti	Sc	TiB, Sc, Ti_6Si_2B, Sc_5Si_3
Ti	Y	TiB, Y, Ti_2B, Y_5Si_3
Ti	Zr	TiB, Ti, Zr_2Si, Ti_6Si_2B
Ti	Hf	TiB, Ti, Hf_2Si, Ti_6Si_2B
Ti	V	TiB, Ti_6Si_2B, V_3B_2, Ti
Ti	Nb	TiB, Ti_6Si_2B, Nb, Nb_5SiB_2
Ti	Ta	TiB, Ti_6Si_2B, Ta_3B_2, Ti
Ti	Cr	TiB, $CTiCr_2$, Ti_5Si_3, Ti_3B_4
Ti	Mo	TiB, Mo, Ti_5Si_3, Ti_6Si_2B
Ti	W	TiB, W, Ti_5Si_3, Ti_6Si_2B
Ti	Mn	TiB, $TiMn_2$, Ti5Si3, Ti3B4
Ti	Fe	TiFe, Ti_5Si_3, TiB_2, Ti_3B_4
Ti	Co	TiCo, Ti_5Si_3, TiB_2, Ti_3B_4
Zr	Sc	Zr_2Si, ZrB_2, Zr, Sc
Zr	Y	Zr_2Si, ZrB_2, Zr, Y
Zr	Ti	Zr, Zr_2Si, ZrB_2, Ti_3B_4
Zr	Hf	Zr, Zr2Si, HfB_2
Zr	V	Zr, Zr_2Si, ZrB_2, V_3B_2
Zr	Nb	Zr, Zr_2Si, ZrB_2, Nb_3B_2
Zr	Ta	Zr, Zr_2Si, TaB, ZrB_2
Zr	Cr	Zr_2Si, ZrB_2, $ZrCr_2$, Zr
Zr	Mo	Zr_2Si, ZrB_2, $ZrMo_2$, Zr_9Mo_4B
Zr	W	ZrB_2, Zr_2Si, Zr, W
Zr	Mn	ZrB_2, Zr_2Si, Zr, Mn
Zr	Fe	ZrB_2, Zr_2Si, Zr, Fe
Zr	Co	ZrB_2, Zr_2Si, Zr, Co
Hf	Sc	Hf_2Si, HfB_2, Hf, Sc
Hf	Y	Hf_2Si, HfB_2, Hf, Y
Hf	Ti	Hf, TiB, Hf_2Si, HfB_2
Hf	Zr	Hf, HfB_2, Hf_2Si, Zr_2Si
Hf	V	Hf, Hf_2Si, HfB_2, V_3B_2
Hf	Nb	Hf, Hf_2Si, HfB_2, Nb_3B_2
Hf	Ta	Hf, Hf_2Si, HfB_2, Ta_3B_2
Hf	Cr	Hf, Cr, HfB_2, Hf_2Si
Hf	Mo	Hf_2Si, HfB_2, $HfMo_2$, Hf_9Mo_4B
Hf	W	HfB_2, Hf_2Si, HfW_2, Hf
Hf	Mn	HfB_2, Hf_2Si, $HfMn_2$, Hf
Hf	Fe	HfB_2, Hf_2Si, $HfFe_2$, Hf
Hf	Co	HfCo, Hf_2Si, HfB_2
V	Sc	V_3B_2, V_5SiB_2, Sc_5Si_3, Y
V	Y	V_3B_2, V_5SiB_2, Y_5Si_3, Y
V	Ti	V_3B_2, V_5SiB_2, Ti_5Si_3, Y
V	Zr	V_3B_2, V_5SiB_2, Zr_2Si
V	Hf	V_3B_2, V_5SiB_2, Hf_2Si
V	Nb	V_5SiB_2, Nb_5SiB_2
V	Ta	V_5SiB_2, Ta_5SiB_2
V	Cr	VB, V_5SiB_2, Cr_3Si
V	Mo	VB, V_5SiB_2, Mo_3Si
V	W	VB, W, V_5SiB_2, V_5Si_3
V	Mn	VB, VMn_2Si, V_5SiB_2
V	Fe	VB, V_5SiB_2, Fe_2VSi
V	Co	V_5SiB_2, VB, Co_2Si, Co
Nb	Sc	Nb_5SiB_2, Nb_3B_2, Sc_5Si_3, Sc
Nb	Y	Nb_5SiB_2, NbB, Y5Si3, Y
Nb	Ti	Nb_5SiB_2, TiB, Ti_6Si_2B, Nb
Nb	Zr	Nb_5SiB_2, Nb3B2, Zr_2Si
Nb	Hf	Nb_5SiB_2, Nb3B2, Hf_2Si
Nb	V	Nb_5SiB_2, V_5SiB_2
Nb	Ta	Nb_5SiB_2, Ta_5SiB_2
Nb	Cr	NbB, Cr, Nb_45Si_3, Nb_3B_2
Nb	Mo	NbB, Mo, Nb_5SiB_2, Nb_5Si_3
Nb	W	NbB, W, Nb_5SiB_2, Nb_5Si_3
Nb	Mn	NbB_2, $NbMn_2$, Nb_5Si_3, NbMnSi
Nb	Fe	NbB, NbFeSi, Nb_5SiB_2, $NbFe_2$
Nb	Co	NbB, Co, Nb5SiB2, Nb_5Si_3
Ta	Sc	Ta_5SiB_2, Ta_3B_2, Sc_5Si_3, Sc
Ta	Y	Ta_5SiB_2, Ta_3B_2, Y_5Si_3, Y
Ta	Ti	Ta_5SiB_2, Ta_3B_2, Ti_6Si_2B, Ti_5Si_3
Ta	Zr	Ta_5SiB_2, Ta_3B_2, Zr_2Si
Ta	Hf	Ta_5SiB_2, Ta_3B_2, Hf_2Si
Ta	V	Ta_5SiB_2, V_5SiB_2
Ta	Nb	Ta_5SiB_2, Nb_5SiB_2
Ta	Cr	TaB, Ta_5SiB_2, Cr_3Si
Ta	Mo	TaB, Ta_5SiB_2, Mo_3Si
Ta	W	TaB, W, Ta5Si3, Ta5SiB2
Ta	Mn	TaB, TaMnSi, Ta_5SiB_2, $TaMn_2$
Ta	Fe	TaB, Ta_5SiB_2, TaFeB, TaFeSi
Ta	Co	TaB, TaCoSi, $CoTa_2$, Ta_5SiB_2
Cr	Sc	Cr_3Si, Cr_5B_3, ScB_2, Sc_5Si_3
Cr	Y	Cr_5B_3, Cr_3Si, Y_5Si_4, Y_5Si_3
Cr	Ti	Cr_3Si, TiB_2, $TiCr_2$, Cr_5B_3
Cr	Zr	Cr_3Si, ZrB_2, Cr
Cr	Hf	HfB_2, Cr_3Si, Cr
Cr	V	VB, CrB, Cr_3Si
Cr	Nb	CrB, Cr_3Si, NbB
Cr	Ta	CrB, Cr_3Si, TaB
Cr	Mo	CrB, Cr_3Si, Mo_2CrB_2, Mo_5Si_3
Cr	W	CrB, Cr_3Si, W_5SiB_2
Cr	Mn	CrB, Cr_3Si, Mn_2B, MnSi
Cr	Fe	CrB, Cr_3Si, Fe_2Si, Cr_5B_3
Cr	Co	Cr_5B_3, CoSi, Cr_3Si, Co_2Si
Mo	Sc	Mo, MoB, ScB_2, $Sc_2Mo_3Si_4$
Mo	Y	MoB, Mo, Mo_5SiB_2, Y_5Si_3
Mo	Ti	Mo_3Si, TiB_2, Mo
Mo	Zr	Mo, ZrB_2, ZrMoSi
Mo	Hf	Mo_3Si, HfB_2, Mo
Mo	V	VB, Mo_5SiB_2, Mo_3Si
Mo	Nb	NbB, Mo_5SiB_2, Mo_3Si
Mo	Ta	TaB, Mo_5SiB_2, Mo_3Si
Mo	Cr	Mo_2CrB_2, Mo_5Si_3, Mo_3Si
Mo	W	MoB, W, Mo_5SiB_2, Mo_3Si
Mo	Mn	Mo_2MnB_2, Mo_5Si_3, Mo_3Si
Mo	Fe	Mo_2FeB_2, FeSi, Mo_3Si, Mo_5SiB_2
Mo	Co	Mo_2CoB_2, Mo5Si3, $MoSi_2$
W	Sc	W_2B, W_5SiB_2, Sc_5Si_3, ScB_2
W	Y	W_2B, W_5SiB_2, WB, Y_5Si_4
W	Ti	W, TiB_2, WSi_2
W	Zr	W, ZrSi, ZrB_2, W_5SiB_2
W	Hf	W, HfB_2, WSi_2
W	V	W, VB, W_5SiB_2, WSi_2
W	Nb	W_5SiB_2, W_2B, NbB, Nb_5Si_3
W	Ta	W, TaB, $W5_SiB_2$, WSi_2
W	Cr	W_5SiB_2, CrB, Cr_3Si
W	Mo	W_2B, $MoSi_2$, Mo_5SiB_3
W	Mn	W_2B, MnSi
W	Fe	W_2B, FeSi
W	Co	W_2B, CoSi
Mn	Sc	Mn_2B, MnSi, Sc_5Si_3, ScB_2
Mn	Y	Mn_2B, MnB, MnSi, Y5Si4
Mn	Ti	Mn_3Si, TiB2, Mn2B, TiMn2
Mn	Zr	Mn_2B, ZrSi
Mn	Hf	Mn_2B, Mn_5Si_3, HfMnSi, HfB_2
Mn	V	Mn_2B, MnSi, VB, VMn_2Si
Mn	Nb	Mn_2B, MnSi, NbMnSi, NbB
Mn	Ta	Mn_2B, MnSi, TaMnSi, TaB
Mn	Cr	Mn_2B, MnSi, CrB, Cr_3Si
Mn	Mo	Mn_2B, MnSi, Mo_2MnB_2, MnSi
Mn	W	Mn_2B, MnSi, W_2B
Mn	Fe	Mn_2B, FeSi
Mn	Co	Mn_2B, CoSi
Fe	Sc	Fe, Fe_2Si, ScB_2
Fe	Y	Fe_2B, FeB, FeSi, Y_5Si_4
Fe	Ti	Fe, Fe_2Si, TiB_2
Fe	Zr	Fe, Fe_2Si, ZrB_2
Fe	Hf	Fe_2B, HfB_2, Fe_2Si, $Hf_6Fe_{16}Si_7$
Fe	V	VB, Fe_2B, Fe_2Si
Fe	Nb	Fe_2B, FeSi, NbFeSi, NbB
Fe	Ta	Fe_2B, FeSi, TaFeB
Fe	Cr	Fe_2B, Fe_2Si, CrB
Fe	Mo	Fe_2B, Fe_2Si, FeSi, Mo_2FeB_2
Fe	W	Fe_2B, FeSi, W_2B
Fe	Mn	MnB, Fe_2B, Fe_2Si
Fe	Co	Fe_2B, CoSi
Co	Sc	Co, ScB_2, Co_2Si
Co	Y	CoB, Co, Co_2Si, Y_5Si_3
Co	Ti	Co, Co_2Si, TiB_2
Co	Zr	Co, Co_2Si, ZrB_2
Co	Hf	CoSi, $HfCo_3B_2$
Co	V	VB, CoB, Co_2Si, Co
Co	Nb	CoB, Co_2Si, NbB, Co
Co	Ta	CoB, $Ta_6Co_{16}Si_7$, CoSi, $Ta_3Co_4B_7$
Co	Cr	CoB, Co_2Si, CrB, Co
Co	Mo	Co_2Si, CoB, MoCoB, Co_3B
Co	W	CoB, Co_2Si, W_2B, Co
Co	Mn	CoB, Co_2Si, Mn_2B, Co
Co	Fe	CoB, Co_2Si, Fe_2B, CoFe

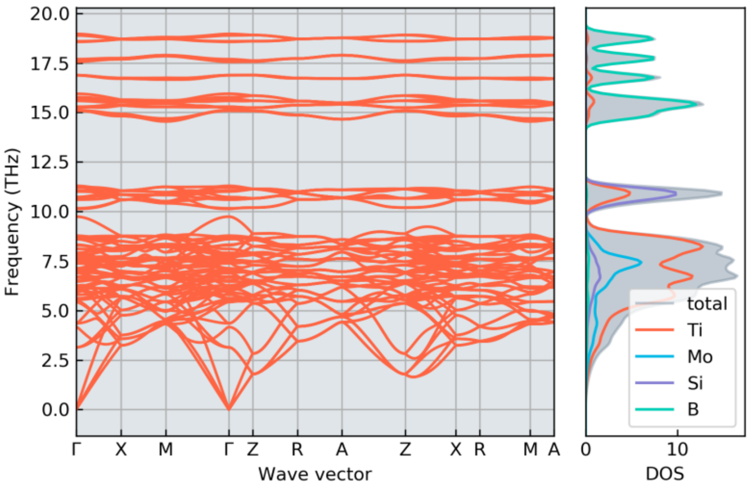

Figure S2. Phonon dispersion and phonon density of states of Ti_4MoSiB_2 indicating its dynamical stability.

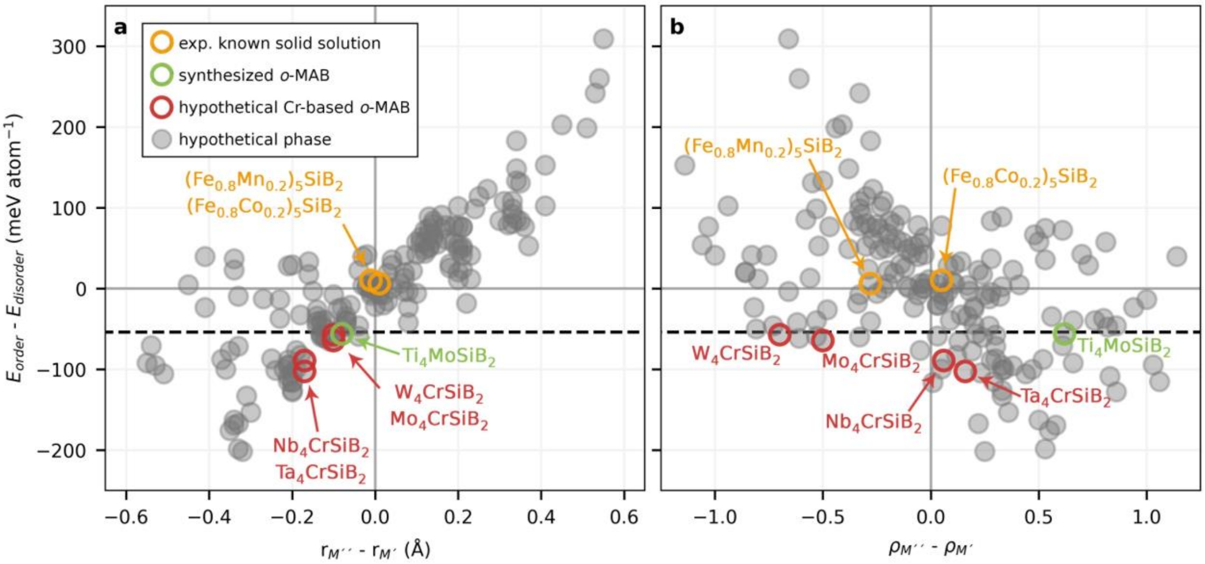

Figure S3. Energy difference between ordered $M'_4M''SiB_2$ and solid solution $(M'_{0.8}M''_{0.2})_5SiB_2$ as function of a) size difference and b) electronegativity difference between M'' and M'. Experimentally realized phases are marked in green for ordered Ti_4MoSiB_2 and in orange for solid solution $(Fe_{0.8}M''_{0.2})_5SiB_2$ (M'' = Mn, Co). In addition, selected hypothetical ordered M'_4CrSiB_2 predicted stable are marked in red. Horizontal dashed line corresponds to an energy difference of zero when entropy contribution at 2000 K to the free energy is considered for solid solution $(M'_{0.8}M''_{0.2})_5SiB_2$.

S2. Characterization of Ti_4MoSiB_2 and *d*-TiO_xCl_y

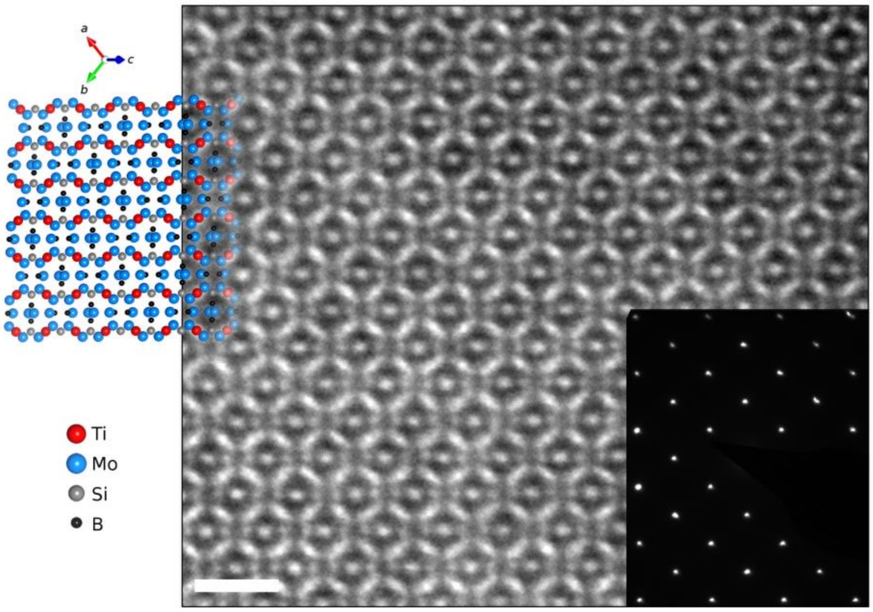

Figure S4. STEM image along the [111] zone axis with corresponding selected area electron diffraction (SAED) for of Ti_4MoSiB_2. Projected schematic to the left represents the corresponding atomic arrangements predicted by DFT. The scale bar corresponds to 1 nm

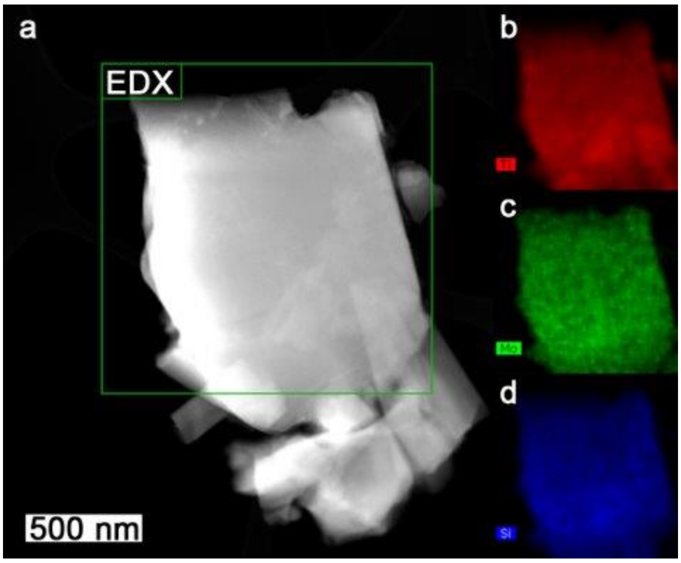

Figure S5. a) EDX mapping of Ti_4MoSiB_2 crystal during TEM analysis along with elemental mapping of b) Ti, c) Mo, and d) Si.

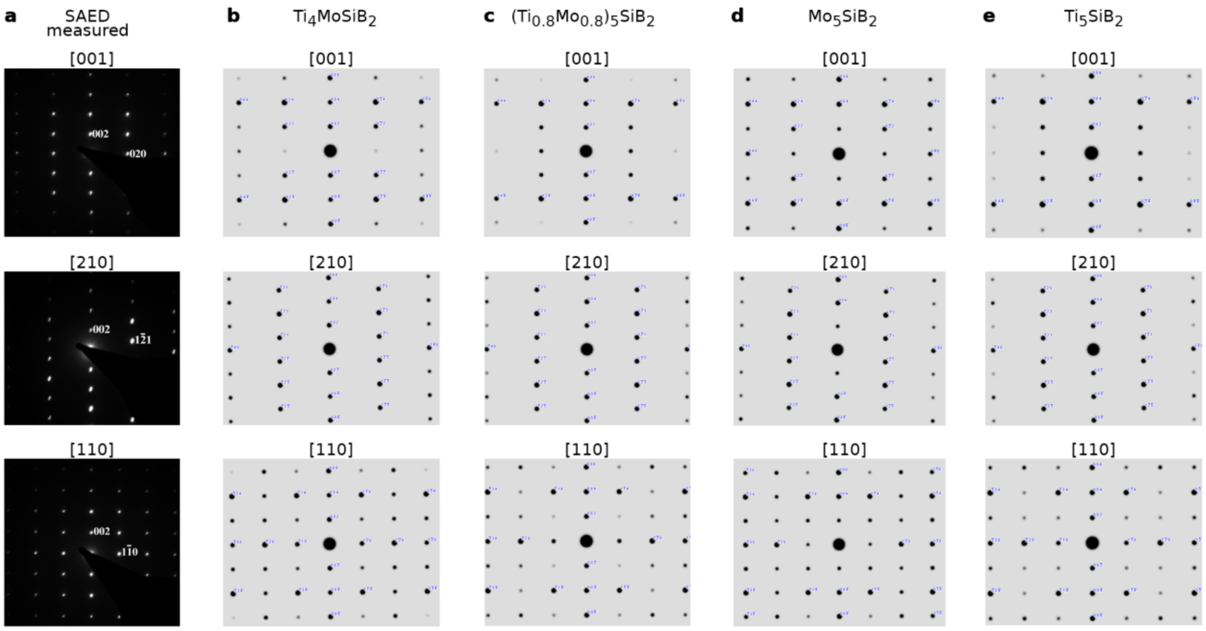

Figure S6. Measured SAED of the Ti_4MoSiB_2 sample and simulated electron diffraction pattern for ordered Ti_4MoSiB_2 (*o*-MAB), solid solution $(Ti_{0.8}Mo_{0.2})_5SiB_2$, Mo_5SiB_2 and Ti_5SiB_2 along three directions.

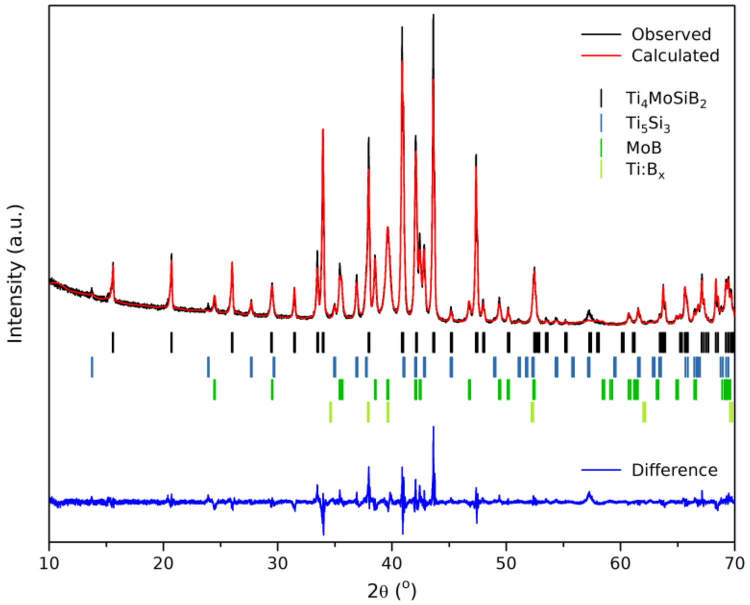

Figure S7. Rietveld refinement of XRD of sample with nominal composition Ti_4MoSiB_2.

Table S3. Rietveld refinement of Ti_4MoSiB_2.

Sample	Ti_4MoSiB_2
Space group	*I*4/*mcm* (140)
χ^2	3.38
R_{wp}	10.0 %
a (Å)	6.06551(3)
b (Å)	6.06551(3)
c (Å)	11.37125(7)
α (°)	90
β (°)	90
γ (°)	90
Ti (16l)	0.67443 (11), 0.17443 (11), 0.13462(8)
Mo (4c)	0.00000(0), 0.00000(0), 0.00000(0)
Si (4a)	0.00000(0), 0.00000(0), 0.25000(0)
B (8h)	0.87507(0), 0.37507(0), 0.00000(0)

Table S4. Calculated crystallographic data for Ti_4MoSiB_2 *o*-MAB using the GGA-PBE exchange-correlation functional with Wyckoff positions given for each unique crystallographic site.

Space group	Lattice parameter (Å)	Atomic position	
*I*4/*mcm* (140)	*a* = 6.094	Ti	16l (0.67618, 0.17618, 0.13157)
	c = 11.436	Mo	4c (0.00000, 0.00000, 0.00000)
		Si	4a (0.00000, 0.00000, 0.25000)
		B	8h (0.89246, 0.39246, 0.00000)

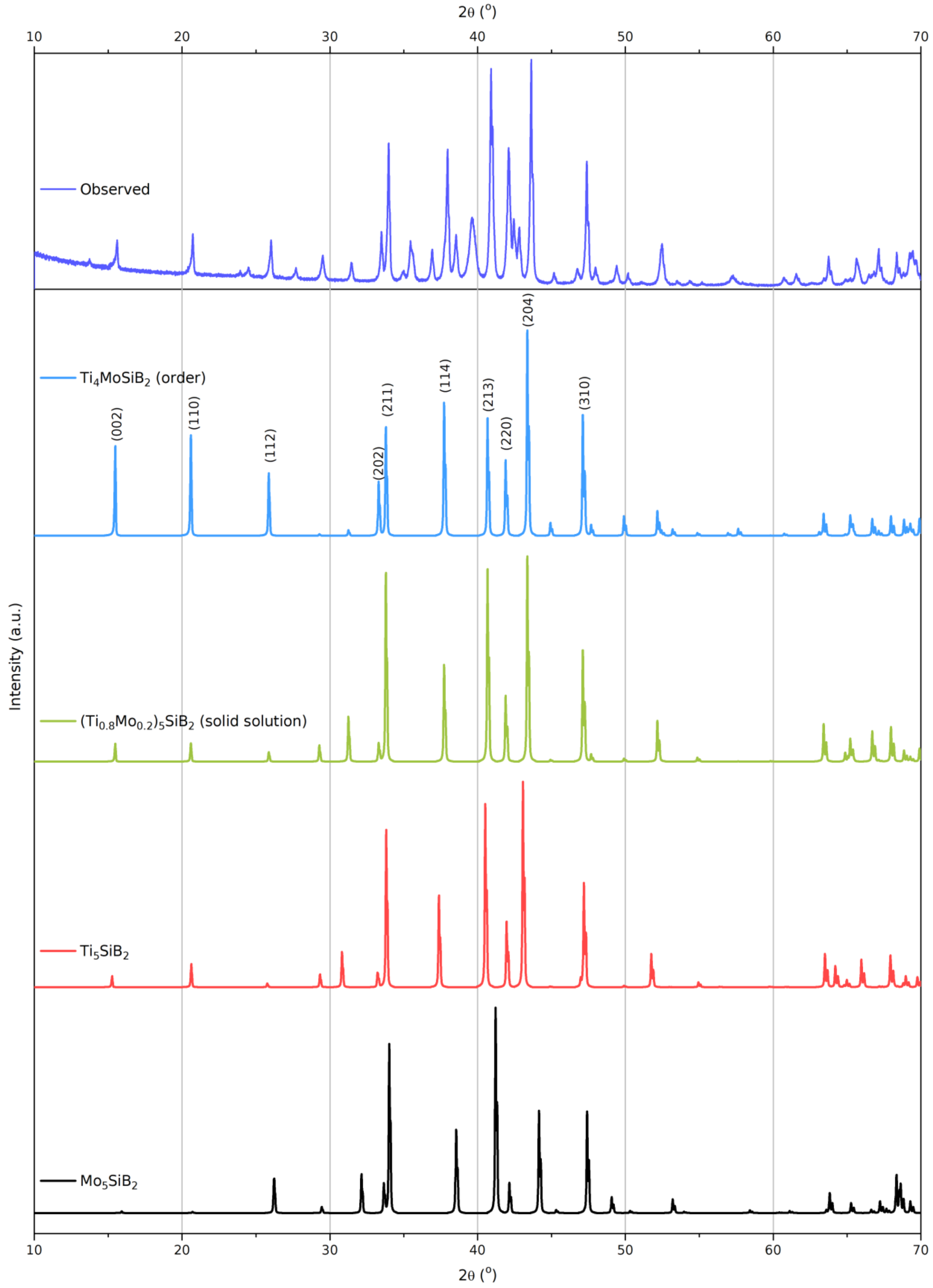

Figure S8 | Measured XRD of the Ti_4MoSiB_2 sample along with simulated diffractograms for ordered Ti_4MoSiB_2 (*o*-MAB), solid solution $(Ti_{0.8}Mo_{0.2})_5SiB_2$, Ti_5SiB_2 and Mo_5SiB_2.

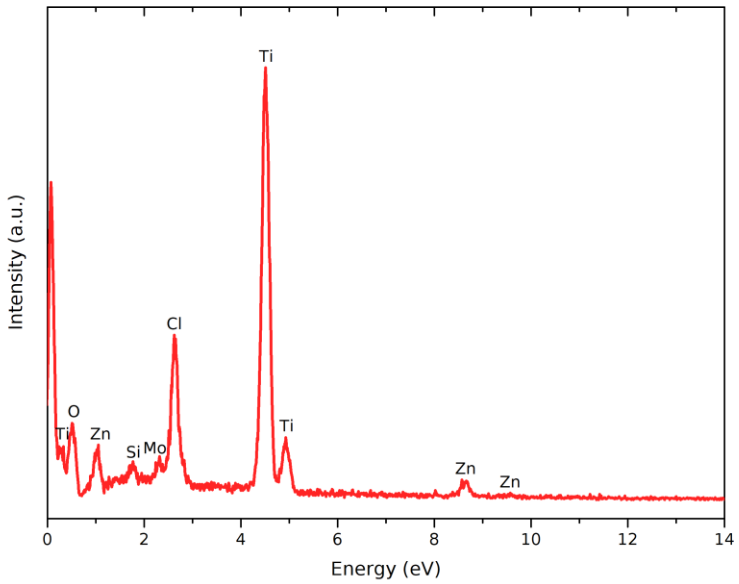

Figure S9. EDX spectrum of the *d*- TiO_xCl_y free-standing film.

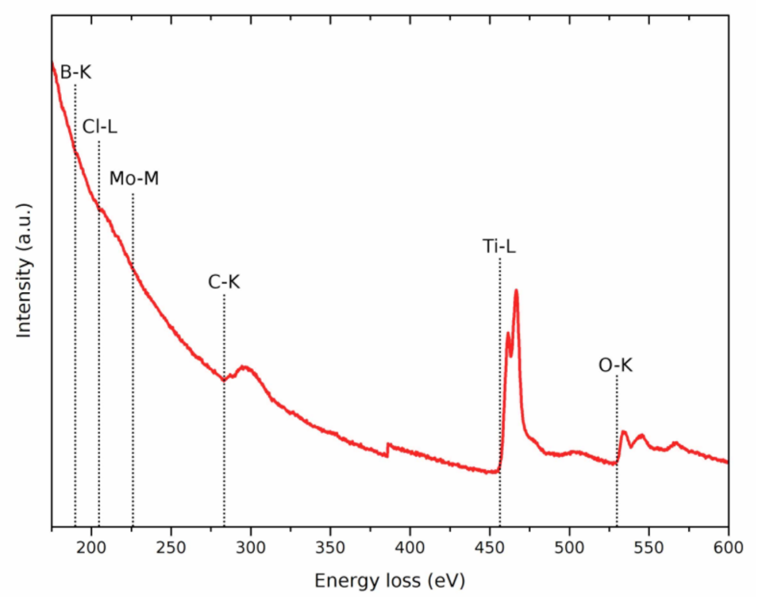

Figure S10. EELS spectrum recorded on single layer *d*-TiO_xCl_y sheet during STEM-EELS analysis.

Table S5. EELS quantification of a single layer *d*-TiO_xCl_y sheet displayed in atomic percent (at. %) for B-K, Cl-L, C-K, Ti-$L_{3,2}$, and O-K edges.

	B at%	Cl at%	Mo at%	C at%	Ti at%	O at%
d-TiO_xCl_y	1.9±0.07	2.18±0.08	0.85±0.08	11.3±0.4	21.7±0.8	62±2

X-ray photoelectron spectroscopy (XPS) measurements performed on cold pressed discs of *o*-MAB (Ti_4MoSiB_2) were used to identify and quantify the elemental composition showing a chemical formula of $Ti_4Mo_{1.2\pm0.1}Si_{1.2\pm0.2}B_{2.5\pm0.4}$ using Ti as a base (global elemental composition is presented in Table S6). The high-resolution spectra for Ti 2*p*, Mo 3*d*, Si 2*p*, B 1*s* and Cl 2*p*

are plotted in Figure S11 and S12. The binding energy (BE) of Ti species in Ti_4MoSiB_2 (Ti 2*p* region) is close to that of Ti metal, TiB_2 and Ti-Mo alloy (±0.2 eV), however it is further from that of $TiSi_x$ (+0.4 eV) (Table S7). As for the BE of Mo species in Ti_4MoSiB_2 (Mo 3*d* region) is most close to Mo metal (+0.4 eV) and Mo-Ti ω phase (+0.3 eV) and further from Mo-Ti ß phase (-0.8 eV), Mo_2B (+0.6 eV) and $MoSi_2$ (+3.4) (Table S8). This means that both Ti and Mo in Ti_4MoSiB_2 have similar chemical bonding to their respective metals and alloys, with exception to Ti which has similar bonding to TiB_2. The BE of Si species in Ti_4MoSiB_2 (Si 2*p* region) is close to that of $TiSi_x$ (+0.3 eV) and far from the BEs of SiO_x (+3.8 eV), Si (+1.3 eV) and $MoSi_x$ (+0.9 eV) (Table S9). However, the BE of B in Ti_4MoSiB_2 (B 1*s* region) is shifted to higher BE than that of TiB_2 (+0.8 eV), Mo-B (+1.3 eV) and B (+2.1) (Table S10).

Calculations of the chemical formula of d-TiO_xCl_y: The chemical formula calculations for *d*-TiO_xCly by multiplying the fraction of each chemical species belonging to TiO_xCly (found in Tables S7 to S12) by their respective element percentage from the global atomic percentages found in Table S6, taking into account to subtract the O species belonging to CO, COO and SiO_x (assuming x =2), the species used in the calculations are:

1. For Ti: Ti-O and Ti-Cl
2. For O: Ti-O, Ti(OH) after subtracting O belonging to CO, COO and SiO_x and $H_2O_{ads.}$
3. For Cl: Ti-Cl

The oxidation state for Ti in *d*-TiO_xCl_y was calculated assuming that the total charge of the compound is zero and the oxidation states of O, OH and Cl are -2, -1 and -1, respectively.

Table S6. Summary of global atomic percentages obtained from the high resolution XPS spectra of the following regions: Ti 2p, Mo 3d, Si 2p, B 1s C 1s, O 1s, Zn 2p, N 1s and Cl 2p, of Ti_4MoSiB_2 cold pressed disc and free-standing film of 2D TiO_xCl_y.

	Ti at%	Mo at%	Si at%	B at%	C at%	O at%	Zn at%	Cl at%	N at%
Ti_4MoSiB_2	16.9±0.3	1.9±0.1	2.8±0.4	8.2±0.9	19.6±1.4	50.4±0.5	NA	NA	NA
d-TiO_xCl_y	6.77±0.1	0.1±0.03	0.8±0.1	<0.1	63.0±0.2	22.6±0.1	0.6±0.1	1.7±0.1	4.5±0.1

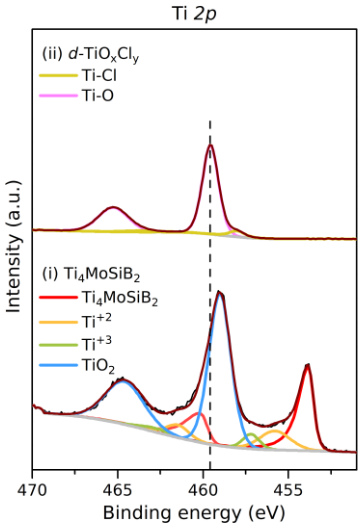

Figure S11. XPS high-resolution spectra with peak fitting of Ti 2p for (i) Ti_4MoSiB_2 and (ii) *d*-TiO_xCl_y. Various peaks shown represent various species assumed to exist. Labels and peak colors are correlated. The results for peak fittings are summarized in Table S6.

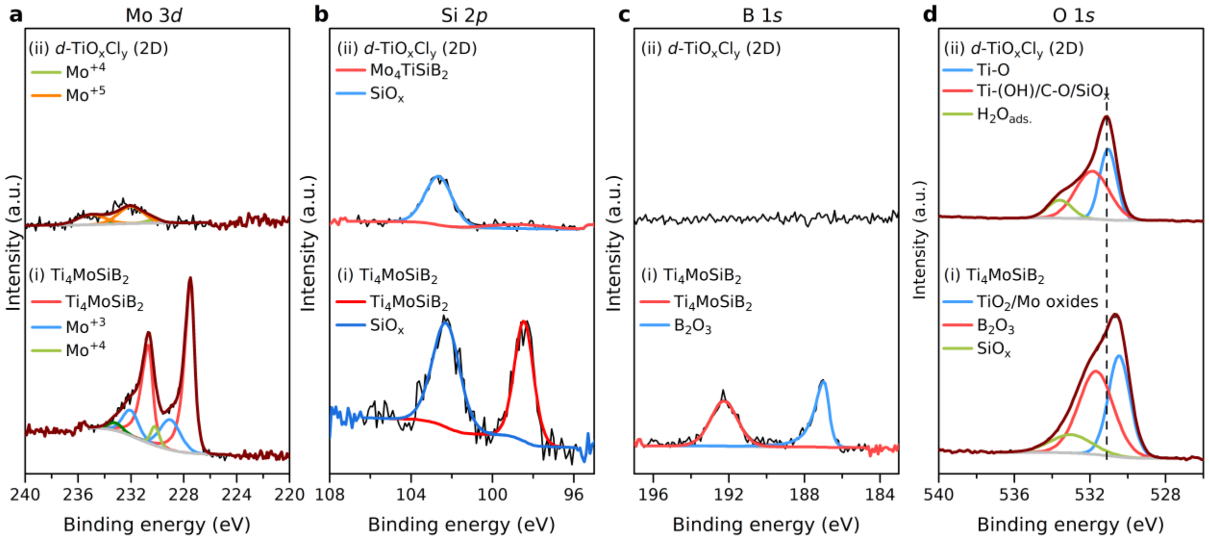

Figure S12. XPS high-resolution spectra with peak fitting of a) Mo 3*d*, b) Si 2*p*, c) B 1*s*, and d) O 1*s* regions for (i) Ti_4MoSiB_2 and (ii) 2D *d*-TiO_xCl_y. Various peaks shown represent various species assumed to exist. Labels and peak colors are correlated. The results for peak fittings are summarized in Tables S6-S11.

Table S7. XPS peak fitting results for Ti 2p region for Ti_4MoSiB_2 cold pressed disc and its 2D TiOCl samples.

Samples	BE [eV][a)]	FWHM [eV][a)]	Fraction	Assigned to	Reference
Ti_4MoSiB_2	453.8(460)	0.8(1.0)	0.27	Ti_4MoSiB_2	[This work]
	453.9±0.3(459.8)			Ti metal	[11]
	454.7			TiB_2	[12]
	453.8(459.9)			Ti-Mo	[13]
	454.2			$TiSi_x$	[14]
	455.8(461.5)	2.1(1.6)	0.10	Ti^{+2}	455.3±0.4(461.1)[11]
	457.2(462.9)	1.1(2.2)	0.04	Ti^{+3}	457.1±0.4(462.7)[11]
	459.0(464.6)	1.5(2.7)	0.59	TiO_2	458.7±0.2(464.4)[11]
d-TiO_xCl_y	458.0(463.7)	1.0(2.2)	0.02	Ti-Cl	[This work]
	458.3(464.4)				$TiCl_4$[15]
	459.6(465.3)	1.1(2.1)	0.98	Ti-O	[This work]

a) Values in parenthesis correspond to the $2p_{1/2}$ peaks. The areal ratios of $2p_{3/2}$ and $2p_{1/2}$ were constrained to 2:1.

Table S8. XPS peak fitting results for Mo 3d region for Ti_4MoSiB_2 cold pressed disc and its 2D TiOCl samples.

Samples	BE [eV][a)]	FWHM [eV][a)]	Fraction	Assigned to	Reference
Ti_4MoSiB_2	227.5 (230.7)	0.8(1.0)	0.27	Ti_4MoSiB_2	[This work]
	227.9±0.1(231.1)			Mo metal	[16]
	226.7(230.1)			Mo-Ti ß phase	[13]
	227.8(231.5)			Mo-Ti ɯ phase	[13]
	228.1			Mo_2B	[17]
	230.9			$MoSi_2$	[18]
	229.0(232.0)	2.1(1.6)	0.10	MoO_2	228.7(231.8)[16]
	230.2(233.2)	1.1(2.2)	0.04	Mo^{+4}	230.0(233.1)[19]
d-TiO_xCl_y	231.1(234.1)	1.3(1.5)	0.4	Mo^{+5}	231.2(234.3)[19]
	232.7(235.7)	1.4(1.5)	0.6	MoO_3	232.6(235.7)[19]

a) Values in parenthesis correspond to the $3d_{5/2}$ peaks. The areal ratios of $3d_{3/2}$ and $2p_{5/2}$ were constrained to 3:2.

Table S9. XPS peak fitting results for Si 2p region for Ti_4MoSiB_2 cold pressed disc and its 2D TiOCl samples.

Samples	BE [eV][a)]	FWHM [eV][a)]	Fraction	Assigned to	Reference
Ti_4MoSiB_2	98.5	1.1	0.45	Ti_4MoSiB_2	[This work]
	102.3	1.6	0.55	SiO_x	102.9±1[20]
	99.8			Si	99.4[20]
	98.8			$TiSi_x$	[14]
	99.4±0.2			$MoSi_x$	[18]
d-TiO_xCl_y	99.0	3.4	0.15	Ti_4MoSiB_2	[This work]
	103.1	1.4(1.5)	0.85	SiO_x	102.9±1[20]

a) Values in parenthesis correspond to the $3d_{5/2}$ peaks. The areal ratios of $3d_{3/2}$ and $2p_{5/2}$ were constrained to 3:2.

Table S10. XPS peak fitting results for B 1s region for Ti_4MoSiB_2 cold pressed disc sample.

Samples	BE [eV]	FWHM [eV]	Fraction	Assigned to	Reference
	186.7	0.6	0.48	Ti_4MoSiB_2	[This work]
	187.5			TiB_2	[21, 22]
Ti_4MoSiB_2	188.0			Mo-B	[18]
	188.8			B	[23]
	192.2	1.7	0.52	B_2O_3	[21]

Table S11. XPS peak fitting results for O 1s region for 2D TiOCl sample.

Samples	BE [eV]	FWHM [eV]	Fraction	Assigned to	Reference
Ti_4MoSiB_2	530.5	1.3	0.38	TiO_2/Mo-oxides	530.2 for TiO_2[21] 530, 530.7, 530.3 eV for Mo-oxides[16, 19]
	531.7	2.2	0.49	B_2O_3	531.7±0.1[24]
	533.0	2.6	0.13	SiO_x	532 to 533[20]
	531.0	1.1	0.37	Ti^{+4}-O	[This work]
d-TiO_xCl_y	531.7	1.9	0.51	C-O/SiO_x/Ti(OH)	532.4±0.2[20, 25]
	533.6	1.5	0.12	$H_2O_{ads.}$	533.2±0.1[26]

Table S12. XPS peak fitting results for Cl 2p region for *d*-TiO_xCl_y.

Samples	BE [eV][a)]	FWHM [eV][a]	Fraction	Assigned to	Reference
d-TiO_xCl_y	199.7(201.4)	1.2(1.1)	1.0	Ti-Cl	[This work]
				$TiCl_4$	[15]

a) Values in parenthesis correspond to the $2p_{1/2}$ peaks. The areal ratios of $2p_{3/2}$ and $2p_{1/2}$ were constrained to 2:1.

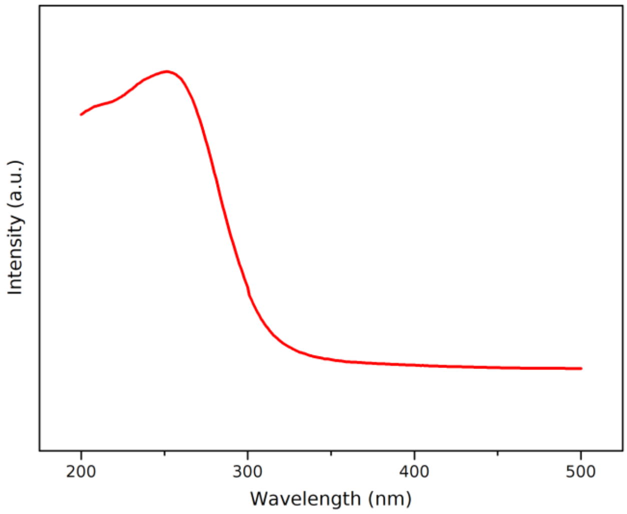

Figure S13. Absorption spectrum of dilute 2D TiO_xCl_y solution.

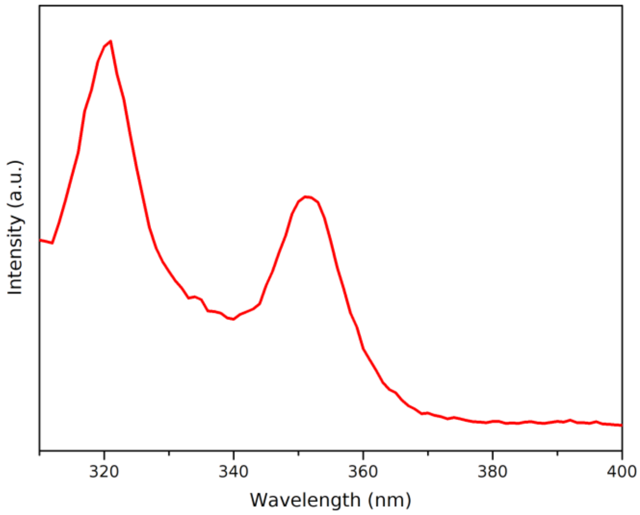

Figure S14. Fluorescence spectra of free-standing TiO_xCl_y film excited at 270 nm.

S3. Electrochemical characterization 2D TiO_xCl_y

The potential of the present 2D TiO_xCl_y in energy storage applications is assessed by constructing a three-electrode Swagelok cell, where 2D *d*-TiO_xCl_y 'paper', activated carbon and Ag/AgCl served as a working, counter, and reference electrode, respectively. The active material loading was ≈ 1.41 mg cm^{-2} and the thickness of free-standing *d*-TiO_xCl_y film was 3.25 µm. Altogether, we use a mass of 100 µg. Figure S15a shows the cyclic voltammograms (CV) of the 2D *d*-TiO_xCl_y 'paper' electrode in 1M H_2SO_4 electrolyte at different scan rates, ranging from 5 mV s^{-1} to 1000 mV s^{-1}. The voltage window was selected by scanning the 2D *d*-TiO_xCl_y electrode for different voltage ranges at 20 mV s^{-1}, as shown in Figure S16, to observes the hydrogen evolution reaction (HER) and oxygen evolution reaction (OER), which are typically characterized by a sharp increase in current at negative and positive sides, respectively. Herein, 2D *d*-TiO_xCl_y was shown to exhibit a combination of redox process and HER beyond -0.1 V (vs. Ag/AgCl), and electrolyte degradation beyond +0.4 V (vs. Ag/AgCl). Therefore, the electrode was scanned in the voltage window of -0.1 to +0.4 V (vs. Ag/AgCl) to avoid irreversible reactions and electrolyte degradation. The non-ideal rectangular shape of the CV curves demonstrates a combination of electrical double-layer and diffusion-controlled charge storage behavior. The corresponding charge/discharge curves (Figure S15be) were measured at different current densities (from 1 to 10 A g^{-1}), which complement the CV results.

Figure S15c shows the variation of gravimetric and volumetric capacitances with the scan rate. The 2D *d*-TiO_xCl_y 'paper' electrode rendered a volumetric and gravimetric capacitance of 275.2 F cm^{-3} and 86 F g^{-1}, respectively, at a scan rate of 5 mV s^{-1}. Interestingly, the 2D *d*-TiO_xCl_y 'paper' electrode rendered excellent rate capability and demonstrated a volumetric capacitance of 185.9 F cm^{-3} at a scan rate of 1000 mV s^{-1}. The superior rate capability indicates potential for rapid charge/discharge applications. These preliminary results, i.e., a volumetric capacitance

of 275 F cm^{-3} at 5 mV s^{-1} and excellent rate capability (185.9 F cm^{-3} at 1000 mV s^{-1}), suggest a potential of 2D TiO_xCl_y for symmetric and asymmetric supercapacitors.

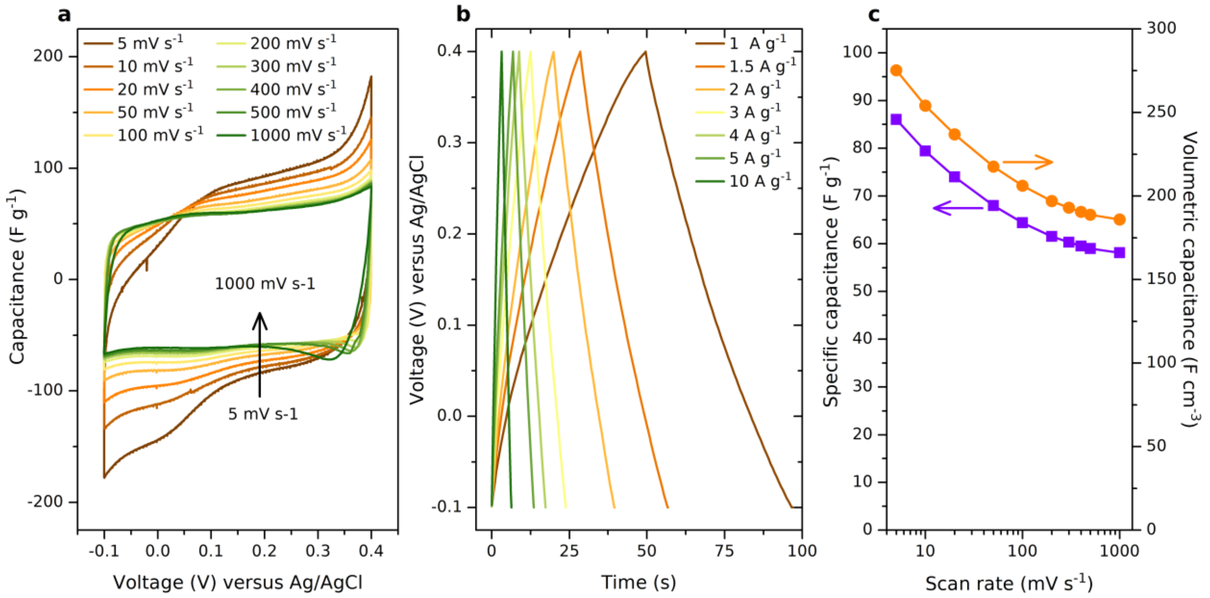

Figure S15. a) Cyclic voltammograms, b) charge/discharge curves, and c) scan rate dependence of a 3.25 µm-thick TiO_xCl_y 'paper' electrode.

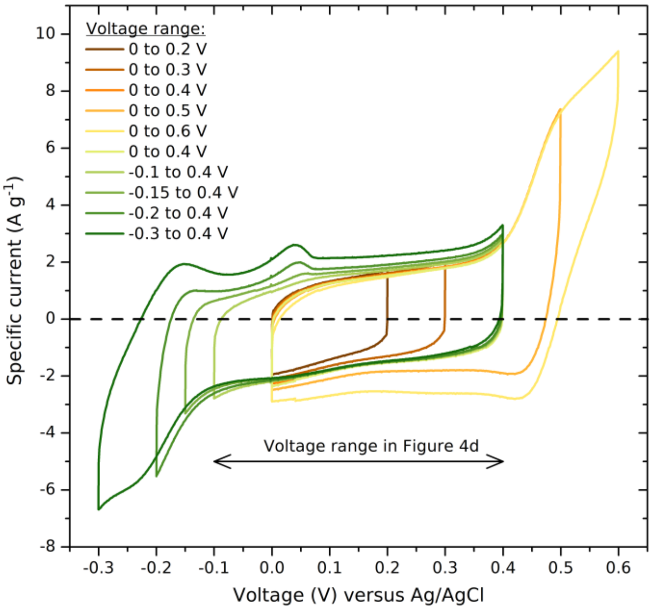

Figure S16. CV curves at 20 mV/s to select the optimal voltage range for 2D TiO_xCl_y electrode

Supplementary References

[1] H. Kudielka, H. Nowotny, G. Findeisen, *Monatsh. Chem.* **1957**, *88*, 1048.
[2] C. A. Nunes, B. B. de Lima, G. C. Coelho, P. A. Suzuki, *J. Phase Equilib. Diff.* **2009**, *30*, 345.
[3] H. Nowotny, F. Benesovsky, E. Rudy, A. Wittmann, *Monatsh. Chem.* **1960**, *91*, 975; G. Rodrigues, C. A. Nunes, P. A. Suzuki, G. C. Coelho, *Intermetallics* **2004**, *12*, 181.
[4] H. Nowotny, B. Lux, H. Kudielka, *Monatsh. Chem.* **1956**, *87*, 447.
[5] H. Nowotny, E. Dimakopoulou, H. Kudielka, *Monatsh. Chem.* **1957**, *88*, 180.
[6] B. Aronsson, *Acta Chem Scand* **1958**, *12*, 31.
[7] M. Fukuma, K. Kawashima, M. Maruyama, J. Akimitsu, *Journal of the Physical Society of Japan* **2011**, *80*, 024702.
[8] B. Aronsson, I. Engström, *Acta Chem Scand A* **1960**, *14*, 1403.
[9] D. M. de Almeida, C. Bormio-Nunes, C. A. Nunes, A. A. Coelho, G. C. Coelho, *J. Magn. Magn. Mater.* **2009**, *321*, 2578.
[10] M. A. McGuire, D. S. Parker, *J. Appl. Phys.* **2015**, *118*, 163903.
[11] M. C. Biesinger, L. W. M. Lau, A. R. Gerson, R. S. C. Smart, *Applied Surface Science* **2010**, *257*, 887.
[12] B. Prakash, E. Richter, H. Pattyn, J. P. Celis, *Surf. Coat. Tech.* **2003**, *173*, 150.
[13] W.-d. Zhang, Y. Liu, H. Wu, M. Song, T.-y. Zhang, X.-d. Lan, T.-h. Yao, *Materials Characterization* **2015**, *106*, 302.
[14] H. Bender, W. D. Chen, J. Portillo, L. Van den Hove, W. Vandervorst, *Applied Surface Science* **1989**, *38*, 37.
[15] C. Mousty‐Desbuquoit, J. Riga, J. J. Verbist, *The Journal of Chemical Physics* **1983**, *79*, 26.
[16] F. Werfel, E. Minni, *J. Phys. C: Solid State Phys.* **1983**, *16*, 6091.
[17] R. Escamilla, E. Carvajal, M. Cruz-Irisson, F. Morales, L. Huerta, E. Verdin, *J. Mater. Sci.* **2016**, *51*, 6411.
[18] P. V. Kiryukhantsev-Korneev, I. V. Iatsyuk, N. V. Shvindina, E. A. Levashov, D. V. Shtansky, *Corros. Sci.* **2017**, *123*, 319.
[19] J. G. Choi, L. T. Thompson, *Applied Surface Science* **1996**, *93*, 143.
[20] J. Finster, D. Schulze, F. Bechstedt, A. Meisel, *Surface Science* **1985**, *152-153*, 1063.
[21] L. Artiglia, D. Lazzari, S. Agnoli, G. A. Rizzi, G. Granozzi, *J. Phys. Chem. C* **2013**, *117*, 13163.
[22] L. Shi, Y. Gu, L. Chen, Z. Yang, J. Ma, Y. Qian, *Inorganic Chemistry Communications* **2004**, *7*, 192.
[23] A. Faussemagne, P. Delichère, N. Moncoffre, A. Benyagoub, G. Marest, *Surf. Coat. Tech.* **1996**, *83*, 70.
[24] W. E. Moddeman, A. R. Burke, W. C. Bowling, D. S. Foose, *Surface and Interface Analysis* **1989**, *14*, 224.
[25] S. Contarini, S. P. Howlett, C. Rizzo, B. A. De Angelis, *Applied Surface Science* **1991**, *51*, 177.
[26] J. Halim, K. M. Cook, P. Eklund, J. Rosen, M. W. Barsoum, *Applied Surface Science* **2019**, *494*, 1138.
